# Integrated meta-analysis and transcriptomics pinpoint genomic loci and novel candidate genes associated with submergence tolerance in rice

**DOI:** 10.1186/s12864-024-10219-z

**Published:** 2024-04-04

**Authors:** Kelvin Dodzi Aloryi, Nnaemeka Emmanuel Okpala, Hong Guo, Benjamin Karikari, Aduragbemi Amo, Semiu Folaniyi Bello, Dinesh Kumar Saini, Selorm Akaba, Xiaohai Tian

**Affiliations:** 1 Hubei Collaborative Innovation Centre for Grain Industry, College of Agriculture, Yangtze University, Jingzhou, China; 2 University of Chinese Academy of Sciences, 100049 Beijing, China; 3 Département de phytologie, Université Laval, Québec, QC Canada; 4 Department of Agricultural Biotechnology, Faculty of Agriculture, Food and Consumer Sciences, University for Development Studies, Tamale, Ghana; 5 Department of Horticultural Sciences, Texas A&M University, College Station, TX USA; 6 Texas A&M AgriLife Research and Extension Center, Weslaco, TX USA; 7 Department of Animal Genetics, Breeding and Reproduction, College of Animal Science, South China Agricultural University, Guangzhou, Guangdong China; 8 Department of Plant Breeding and Genetics, Punjab Agricultural University, Ludhiana, Punjab India; 9 Department of Plant and Soil Science, Texas Tech University, Lubbock, TX USA; 10 School of Agriculture, University of Cape Coast, Cape Coast, Ghana

**Keywords:** Meta-analysis, MQTL, Submergence tolerance, Haplotype analysis, Differentially expressed candidate genes (DECGs), Marker-assisted breeding

## Abstract

**Background:**

Due to rising costs, water shortages, and labour shortages, farmers across the globe now prefer a direct seeding approach. However, submergence stress remains a major bottleneck limiting the success of this approach in rice cultivation. The merger of accumulated rice genetic resources provides an opportunity to detect key genomic loci and candidate genes that influence the flooding tolerance of rice.

**Results:**

In the present study, a whole-genome meta-analysis was conducted on 120 quantitative trait loci (QTL) obtained from 16 independent QTL studies reported from 2004 to 2023. These QTL were confined to 18 meta-QTL (MQTL), and ten MQTL were successfully validated by independent genome-wide association studies from diverse natural populations. The mean confidence interval (CI) of the identified MQTL was 3.44 times narrower than the mean CI of the initial QTL. Moreover, four core MQTL loci with genetic distance less than 2 cM were obtained. By combining differentially expressed genes (DEG) from two transcriptome datasets with 858 candidate genes identified in the core MQTL regions, we found 38 common differentially expressed candidate genes (DECGs). *In silico* expression analysis of these DECGs led to the identification of 21 genes with high expression in embryo and coleoptile under submerged conditions. These DECGs encode proteins with known functions involved in submergence tolerance including WRKY, F-box, zinc fingers, glycosyltransferase, protein kinase, cytochrome P450, PP2C, hypoxia-responsive family, and DUF domain. By haplotype analysis, the 21 DECGs demonstrated distinct genetic differentiation and substantial genetic distance mainly between *indica* and *japonica* subspecies. Further, the MQTL7.1 was successfully validated using flanked marker S2329 on a set of genotypes with phenotypic variation.

**Conclusion:**

This study provides a new perspective on understanding the genetic basis of submergence tolerance in rice. The identified MQTL and novel candidate genes lay the foundation for marker-assisted breeding/engineering of flooding-tolerant cultivars conducive to direct seeding.

**Supplementary Information:**

The online version contains supplementary material available at 10.1186/s12864-024-10219-z.

## Background

Rice (*Oryza sativa* L.) remains the most-eaten staple food for over half of the global population [1]. By 2025, the global rice demand is expected to increase by 25%, which requires a production target of 732.5 MT, with an annual increase in production of 5.9 MT [2, 3]. The irrigated rice ecosystem accounts for 55% of cultivated areas and provides 75% of the world’s rice production [4]. However, the current trend in changing climates has further exacerbated the decline in rice production worldwide [5]. The practice of transplanting rice seedlings into puddled fields is widely adopted in most rice production areas in the world. Currently, direct seeding under rainfed and irrigated conditions is preferred by farmers due to the low cost of labour and convenience [6–8]. Farmers usually flood direct-seeded fields to suppress weed growth, thus reducing manual weeding and excessive herbicide use [3, 9]. However, this practice creates stress associated with submergence [10]. Rice is highly sensitive to anoxic conditions during seed germination, thus commercializing direct seeding requires breeding of rice varieties with increased submergence tolerance [11].

To withstand anoxic stress during seed germination, rice typically adopts two distinct strategies such as escape and quiescence [12]. The quiescence approach is mainly associated with the elongation of shoot by *SUB1* and survival by limiting carbohydrate consumption [13, 14]. On the other hand, the escape strategy enhanced rapid elongation of coleoptile and mesocotyl to access the water surface and transport oxygen to submerged tissues [15, 16]. Consequently, the success of seedling establishment depends on the length and the speed of coleoptile and mesocotyl elongation. However, the genetic basis of this event is less established. A considerable number of quantitative trait loci (QTL) controlling submergence germination have been identified in the past few years [17–22]. These studies detected numerous QTL in diverse mapping populations with distinct marker types [17–24]. However, there are inherent difficulties associated with the implementation of these QTL in breeding programs. The conventional QTL mapping method could lead to a wide confidence interval (CI) and undetermined QTL location. In addition, factors including mapping populations, experimental conditions, QTL mapping models, marker density, and environment affect QTL usage in marker-assisted breeding [25, 26].

To resolve these limitations, a new paradigm in QTL analysis (meta-analysis) approach was introduced [27]. This approach takes into account QTL information from multiple studies and integrates them into a consensus map enabling robust statistical power over a large dataset [28, 29]. Moreover, this method has efficiently reduced the confidence region of MQTL leading to high-resolution marker-assisted breeding and candidate gene (CG) mining [30]. Although meta-QTL analyses have been performed on abiotic-related stress and MQTL loci associated with salt, drought, cold stress, and metal ion tolerance have been identified in rice [31, 32, 33], no MQTL information is available on submergence tolerance in rice during germination. Moreover, the advent of high-throughput technology based on SNP arrays and the use of genome-wide association studies (GWAS) to dissect significant genetic loci governing complex quantitative traits is gaining popularity [34]. The available evidence has demonstrated that GWAS and MQTL could be combined to effectively explore important genomic loci and decipher genetic underlying key quantitative traits [35].

Herein, we aimed to perform a meta-QTL analysis of QTL regulating rice submergence tolerance identified in recent years and incorporate GWAS and transcriptome studies to dissect the genomic regions and potential candidate genes underpinning submergence tolerance during germination. The results from this study would aid MQTL-assisted breeding for rice cultivars suitable for direct seeding. In addition, the current study provides baseline information for understanding the metabolic processes underlying submergence tolerance or susceptibility in rice.

## Methods

### Screening of submergence-related traits QTL

A comprehensive search for QTL underlying submergence tolerance in rice was conducted on recently published articles from 2004 to 2023 and a total of 16 QTL studies were obtained that could furnish the initial information required for meta-analysis. The salient information about these QTL are summarized in Table 1. For each QTL study, the submergence-related traits were mainly anaerobic germination (AG), submergence (SUB), low-temperature germination (LTG), and lowest elongated internode (LEI). The initial information collected on each QTL included: (i) size of the mapping population; (ii) associated traits; (iii) logarithm of odd (LOD) score; (iv) phenotypic variance explained (PVE) by initial QTL; (v) closely linked molecular markers; and (vi) type of mapping population (F_2_, Backcross, and recombinant inbred lines (RILs)). The submergence-associated QTL in each study with phenotypic variance explained (PVE) values less than 10% were retained for subsequent analyses. For QTL with missing LOD and PVE score, 3 and 10% were used according to Venske et al. [36] and Khahani et al. [37]. In addition, the 95% CI of each QTL was recalculated based on the type and size of the mapping population following the standardized formula: (1) F_2_ and backcross population, CI = 530/ (Mapping population × R^2^); (2) RIL, CI = 163/ (Mapping population x R^2^). Where R^2^ represents the PVE by individual QTL and the mapping population was the number of lines used in the initial mapping studies [26, 38]. The details of these initial QTL are presented in Supplementary Table 1.

**Table 1:** Summary of QTL studies utilized for meta-QTL analysis

Parents	Population type^a^	Population size	No. of markers	Marker type^b^	Traits^c^	References
IR64/KHAIYAN//IR64	BC_2_	150	155	SSR	AG	[92]
IR64/Khao Hlan	BC_2_	398	136	SSR	AG	[22]
IR64 x Nanhi	F_2_	300	234	SNP	AG	[93]
IR64 x Kharsu80A	F_2_	190	384	SNP	AG	[11]
BJ1 x NSIC Rc222	BC_2_	205	102	SSR	AG	[19]
FR13A x IR42	RIL	103	232	SSR, SNP, STS	SUB	[18]
IR42 x FR13A	RIL	103	232	SNP, SSR, STS	AG	[18]
Kinmaze (japonica) x DV85 (indica)	RIL	81	137	RFLP	AG	[94]
USSR5 x N22	F_2_	148	121	SSR	LTG, AG	[95]
Tai Nguyen x Anda.	F_2_	285	1405	SNP	AG	[96]
Kinmaze (japonica)/DV85 (indica)	RIL	81	137	RFLP, SSR	AG	[97]
Indra x AC 39,416 A	RIL	184	104	SSR	AG	[21]
Swarna Sub1 x AC39416A	F_2_	188	83	SSR	AG	[20]
IR72 x Madabaru	F_2_	466	115	SSR	SUB	[98]
IR42 x Ma-Zhan Red	F_2_	175	118	SSR	AG	[99]
Habiganj x Patnai23	F_2_	192	85	RFLP, SSR	LEI	[100]

^a^BC: backcross population, F_2_: second filial generation population, RIL: recombinant inbred lines. ^b^SSR: simple sequence repeat, SNP: single nucleotide polymorphism, STS: sequence-tagged sites, RFLP: restriction fragment length polymorphism. ^c^AG: anaerobic germination, SUB: submergence, LTG: low-temperature germination, LEI: lowest elongated internode

### Construction of the consensus map and QTL projection

A high-density consensus map was constructed using the R package *LPmerge* employed in our previously developed reference map [39] and one constructed by Prakash et al. [40]. Also, markers linked to individual QTL were included in the construction of the consensus map to ensure high number of initial QTL is projected. QTL absent on the reference map were eliminated. Based on QTL projection, two approaches were adopted. First, the method proposed by Goffinet and Gerber, [27] was used when the number of QTL present on individual chromosomes is less than 10. Sequel to this, the best model with the lowest Akaike information criterion (AIC) scores in BioMercator (https://versailles.inra.fr/Tools/BioMercator-v4, accessed on 1, 4 2023) was considered. Second, a two-step method proposed by Veyrieras et al. [29] was applied when the number of QTL on each chromosome exceeded 10. In the first stage, QTL on each chromosome were clustered using standard parameters. The MQTL on individual chromosomes were then estimated based on four criteria such as AIC model, corrected AIC (AICc and AIC3) model, Average Weight of Evidence (AWE) model, and Bayesian Information Criterion (BIC) model. The model with the lowest values of these criteria was chosen for the next step of analysis. In the second stage, the 95% CI and MQTL positions were ascertained following the best-selected model in the previous step. The QTL were then integrated to obtain the peak position of the initial QTL involved in MQTL CI [41]. However, QTL that did not meet the minimum AIC threshold were eliminated. MQTL were named according to their genetic positions on the rice chromosomes (i.e. MQTL1.1, MQTL2.1, MQTL3.1). The phenotypic variance explained (PVE) by MQTL was calculated as the mean PVE of initial QTL involved in each MQTL.

### Validation of MQTL by GWAS‑based marker-trait associations (MTAs)

MQTL identified in the current study were mapped to the rice reference genome. The markers flanking individual MQTL were manually searched, and their primer sequences were retrieved from the gramene database (https://archive.gramene.org/, accessed on 14, 4 2023). The flanking marker and primer sequences were blasted against the rice reference genome sequence in phytozome (https://phytozome-next.jgi.doe.gov/, accessed on 15, 4 2023) to obtain the physical coordinates of these markers. For markers without physical coordinates, the physical positions were manually anchored. The marker-trait association (MTA) reported from nine independent GWAS involving submergence-associated traits published from 2015 to 2023 were collected to authenticate the accuracy of MQTL loci. The detailed information on these GWAS is presented in Supplementary Table 2. The phenotypic data of these studies included mini-core collections obtained from 4 different countries with panel sizes ranging from 153 to 498. Physical coordinates of significant SNPs associated with submergence-related traits were collected from respective studies. Finally, the physical coordinates of the MQTL identified in this study were compared with the physical locations of MTA in GWAS. MQTL co-localized with significant single nucleotide polymorphisms (SNPs) were deemed GWAS-validated/verified MQTL.

### Candidate genes (CGs) excavation in core MQTL regions

The Rice Genome Annotation Project (RGAP) database (https://rice.uga.edu/cgi-bin/gbrowser/rice, accessed on 20, 4 2023) was used to retrieve the functional candidate genes at CI of core MQTL. The above database was then used to obtain the description of the CGs in the core MQTL intervals. To enhance the accuracy of our assumptions on CGs, the orthologous genes in the core MQTL regions were placed over the genomes of sorghum and maize. The TBtools software [42] was used to visualize the gene density and orthologue genes in sorghum and maize.

### Validation of CGs in transcriptome datasets and gene expression analysis

A survey of two differentially expressed genes (DEGs) datasets involved in submergence tolerance during seed germination from independent studies [43, 44] was explored to validate candidate genes (CGs) found in core MQTL loci. A Venn diagram was employed to compare the common genes regulating submergence tolerance detected by two DEGs studies, and the genes located in MQTL regions. Venny online repository (https://bioinformatics.psb.ugent.be/webtools/Venn/, accessed on 1, 5 2023) was used to analyze shared candidate genes between MQTL regions, and differentially expressed genes (DEGs) datasets. The shared genes are referred to in this study as differentially expressed candidate genes (DECGs).

### Interaction network analysis, GO and KEGG analysis of DECGs

STRING database (Search Tool for the Retrieval of Interacting Genes) (https://string-db.org/, accessed on 4, 5 2023) [45] was used to construct the protein-protein interaction of DECGs. Functional analysis of DECGs in core MQTL interval was conducted using CARMO Comprehensive Annotation of Rice Multi-Omics tool (http://bioinfo.sibs.ac.cn/carmo/Gene_Annotation.php, accessed on 5, 5 2023). Then Gene ontology (GO) terms of DECGs were visualized using TBtools software [42]. The KOBAS v3.0 software (http://kobas.cbi.pku.edu.cn/, accessed on 5, 5 2023) was employed to map DECGs in Kyoto Encyclopedia of Genes and Genomes (KEGG) pathway. The bioinformatics online tool (http://www.bioinformatics.com.cn, accessed on 5, 5 2023) was exploited to display the KEGG results.

### Expression profiling and promoter analysis of DECGs

*In silico* expression analysis of DECGs was carried out using publicly available transcriptome data deposited in NCBI (https://www.ncbi.nlm.nih.gov/gds/, accessed on 10, 5 2023) with series accession ID GSE136885. The reported transcriptome data [46] including embryo at 2 days and coleoptile at 4 days, 8 days, and 14 days under submerged conditions was then used to study the expression patterns of DECGs. Expression levels of DECGs were assessed by transcripts per million (TPM) scores and visualized using a heatmap of log2 in TBtools [42]. Moreover, the 2000-bp upstream of the translational start site (i.e., ATG) of each potential DECGs was obtained from the phytozome database (https://phytozome-next.jgi.doe.gov/, accessed on 12, 5 2023) and uploaded to PlantCARE (https://bioinformatics.psb.ugent.be/webtools/plantcare/html/, accessed on 13, 5 2023) to predict *cis*-acting regulatory elements (CAREs) present in the potential DECGs, and the result visualized using TBtools [42].

### Haplotype analysis of candidate genes

In order to explore the haplotype diversity of potential DECGs, haplotypes were evaluated in RiceVarMap software v2.0 (https://ricevarmap.ncpgr.cn/vars_in_gene/, accessed on 1, 6 2023). The variations present in the 2 kb upstream, 1 kb downstream, and coding sequence of the potential DECGs were retrieved and filtered to remove SNP markers that possessed the lowest allele frequencies (MAF < 60%). Then SNP markers in these genes were used to construct the haplotype network and distribution of the rice subpopulation.

### Validation of MQTL by flanked marker

The conventional method of QTL validation involves developing near-isogenic or transgenic lines to examine QTL effect. In context, MQTL validation differs from the well-known QTL validation approach due to its inclusive property [47]. In a condensed genomic locus, MQTL identification guarantees the validation of known QTL, and therefore validating flanked markers on genotypes with extreme phenotypes has important breeding implications [27]. The peak RFLP marker linked to the core MQTL7.1 was selected for validation on a set of six rice genotypes. The experimental genotypes were submerged for 7 days and mean coleoptile length was recorded. The genotypes with short coleoptile length were considered sensitive and those with long coleoptile length were regarded as tolerant to submergence. Pure genomic DNA was extracted from young seedlings using the standard Cetyl trimethyl ammonium bromide (CTAB) protocol [48]. The quality and quantity of genomic DNA were determined before thermal amplification of MQTL flanked marker region. The PCR cycling conditions involved an initial denaturation of 2 min at 94 °C, followed by 37 cycles of denaturation at 94 °C for 30 s, annealing at 60 °C for 30 s, extension at 72 °C for 10 s, and a final extension at 72 °C for 25 min. Amplified fragments were visualized using gel electrophoresis. The detailed information on the primers used for MQTL validation is presented in Supplementary Table 9.

## Results

### Characteristics of submerged-related traits in rice

**Fig. 1:**
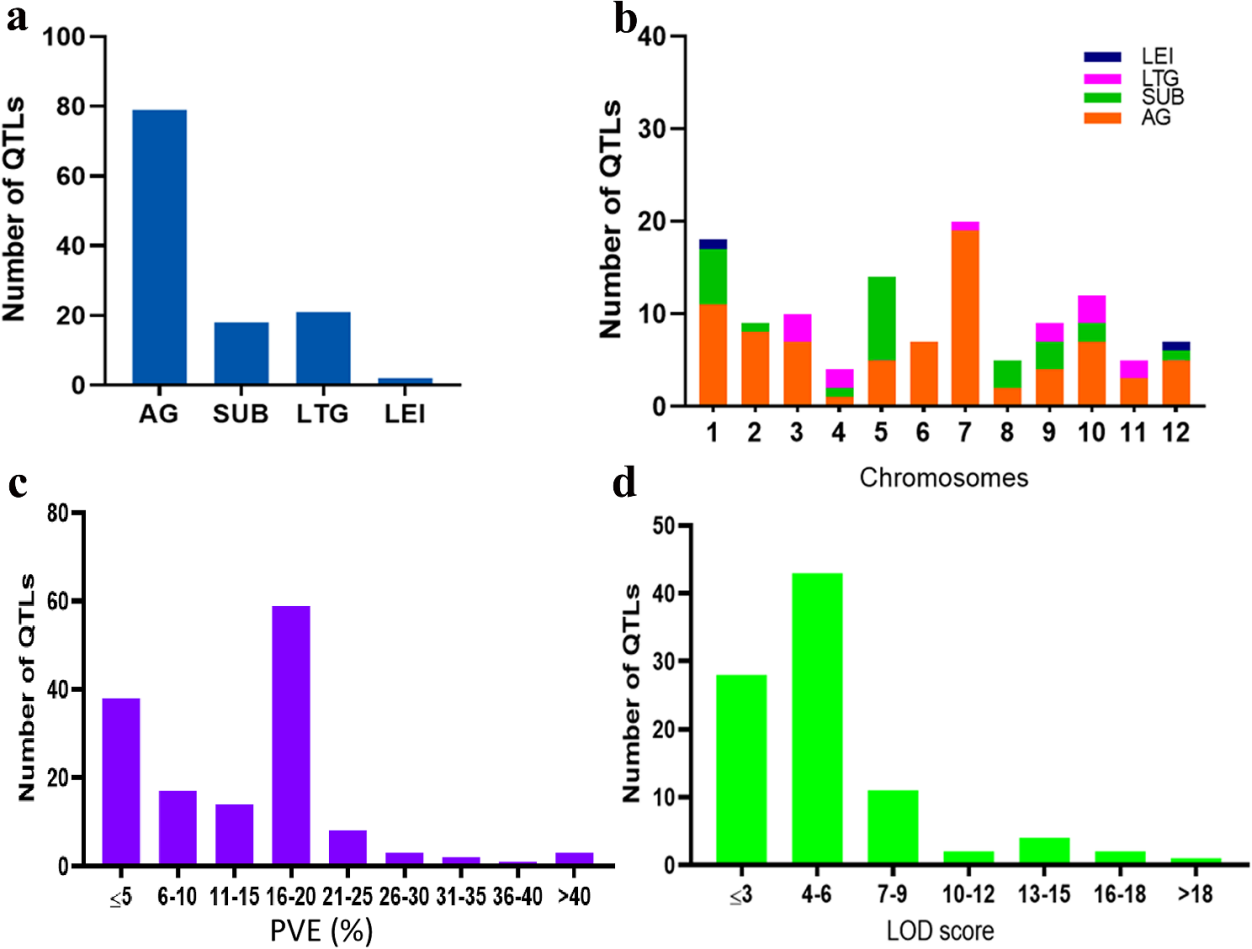
Salient features of the initial quantitative trait loci (QTL) used in this study. **a** Distribution of 121 initial QTL associated with submergence-related traits; AG (anaerobic germination), SUB (submergence), LTG (low-temperature germination), LEI (lowest elongated internode), **b** Trait-wise distribution of QTL on the 12 chromosomes of rice, **c** Distribution of phenotypic variance explained (PVE) by initial QTL, d Logarithm of odds values of the initial QTL

A total of 16 independent studies published from 2004 to 2023 involving eight F_2_ generation, five RILs, and three backcross populations (BC) were assessed to compile QTL information on submergence-related traits (Table 1). Herein, 120 QTL associated with submergence tolerance during germination were collected. Among the studied traits, QTL involving anaerobic germination (65.83%) were most represented followed by low-temperature germination (17.5%), submergence (15.0%), and lowest elongated internode (1.67%) (Fig. 1a). The distribution of QTL on rice chromosomes was uneven, with chromosome 7 accounting for the largest number of QTL (16.67%; 20/120) followed by chromosomes 1 and 5 with 15% (15/120) and 11.67% (14/120), respectively (Fig. 1b). However, chromosome 4 contained the least QTL (3.33%; 4/120). The PVE (%) by each QTL from different studies ranged from 0.09 to 59.08% (Fig. 1c). Interestingly, majority of the initial QTL (53.33%; 64/120) had PVE above the 10% threshold. The LOD scores of initial QTL ranged from 1.35 to 18.2 with a mean of 5.03. The distribution of LOD values of the initial QTL is presented in Fig. 1d.

### Consensus map of rice

**Fig. 2:**
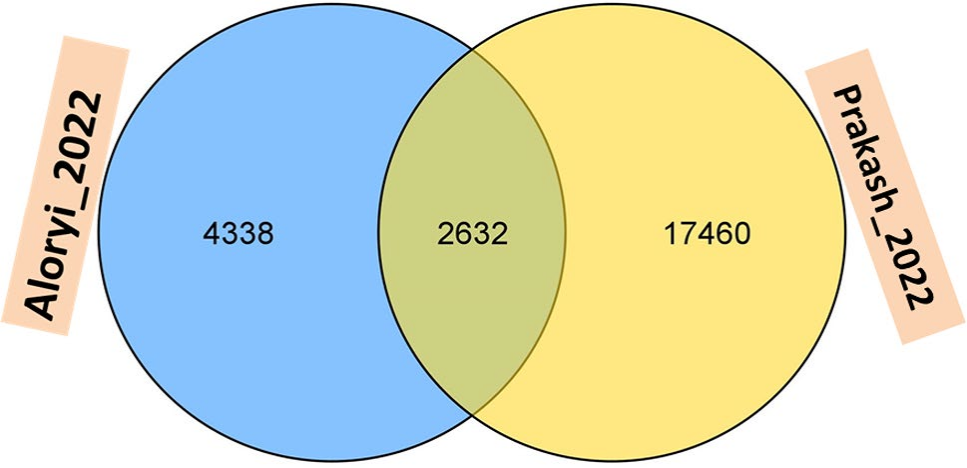
Venn diagram of unique and common markers among the two maps used to construct the consensus map

The consensus map developed in this study consisted of 24,644 markers which included different types of markers such as simple sequence repeat, single nucleotide polymorphism, sequence-tagged sites, and restriction fragment length polymorphism. More importantly, genes including *OsSSII*, *OsSSI*, *Amy1*, and *ACT1* were also integrated into the consensus map. The total length of the reference map was 1,457.74 cM with a mean chromosome length of 1,341.63 cM (Supplementary Fig. 1). Moreover, individual chromosomal distance ranged from 45.71 (on chromosome 7) to 201.97 cM (on chromosome 8). Overall, chromosome 1 displayed the highest number of markers (3,048) and the lowest was chromosome 10 with 1354 markers. The two consensus maps adopted in this study shared 10.8% of markers; Aloryi_2022 had 4,338 unique markers (17.8%) whiles Prakash_2022 had 17,460 unique markers (71.5%) (Fig. 2). Markers on individual linkage groups were sparsely distributed with two ends of the chromosome displaying uneven marker density (Supplementary Fig. 1).

### QTL projection and identification of MQTL

**Fig. 3:**
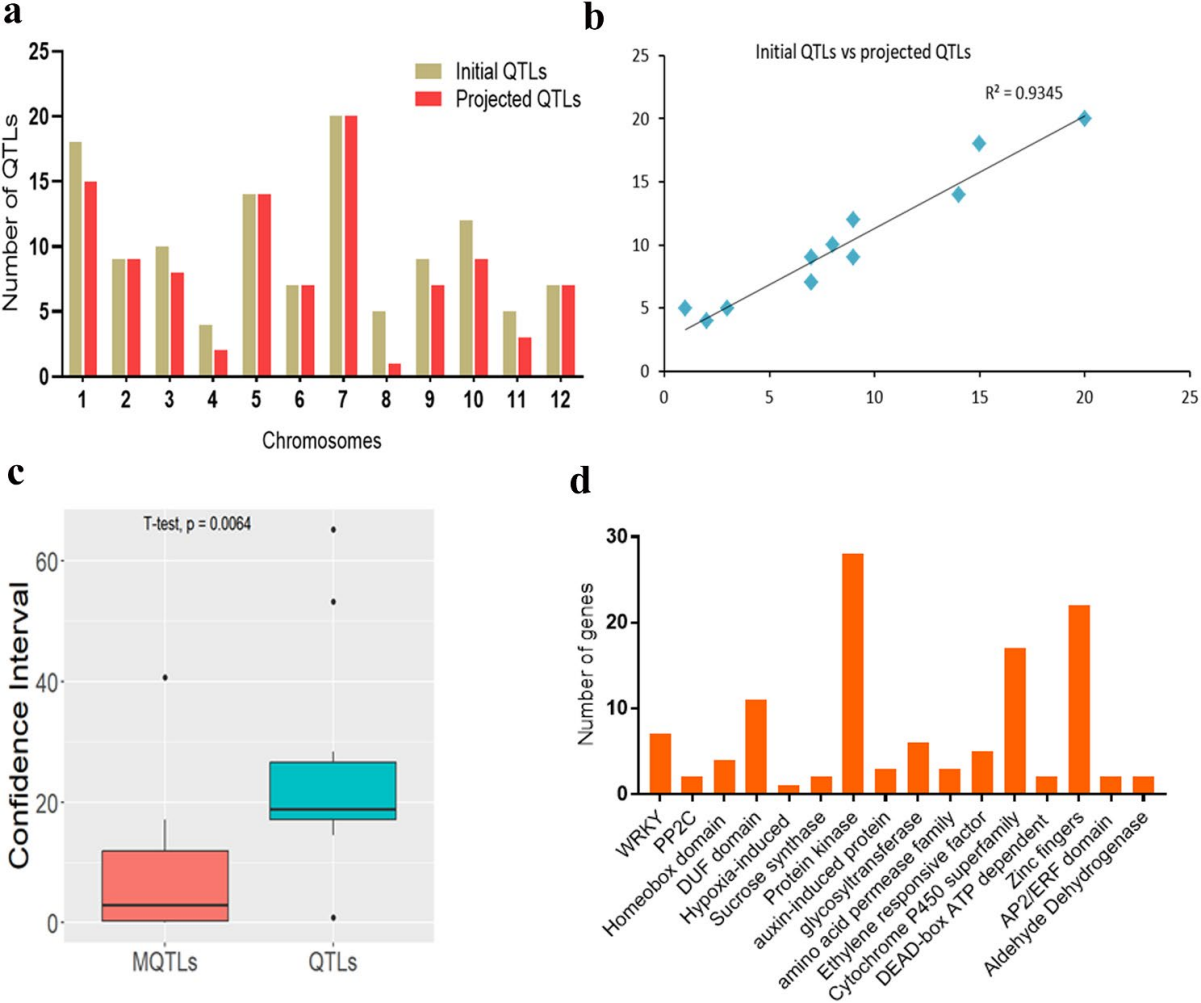
Basic features of initial QTL and Meta-QTL (MQTL) in this study. **a** Frequency of initial QTL and projected QTL, **b** Correlation between initial QTL and projected QTL, **c** Comparison of mean confidence interval (CI) for initial QTL and MQTL, **d** Distribution of candidate genes encoding known protein families associated with submergence tolerance

**Fig. 4:**
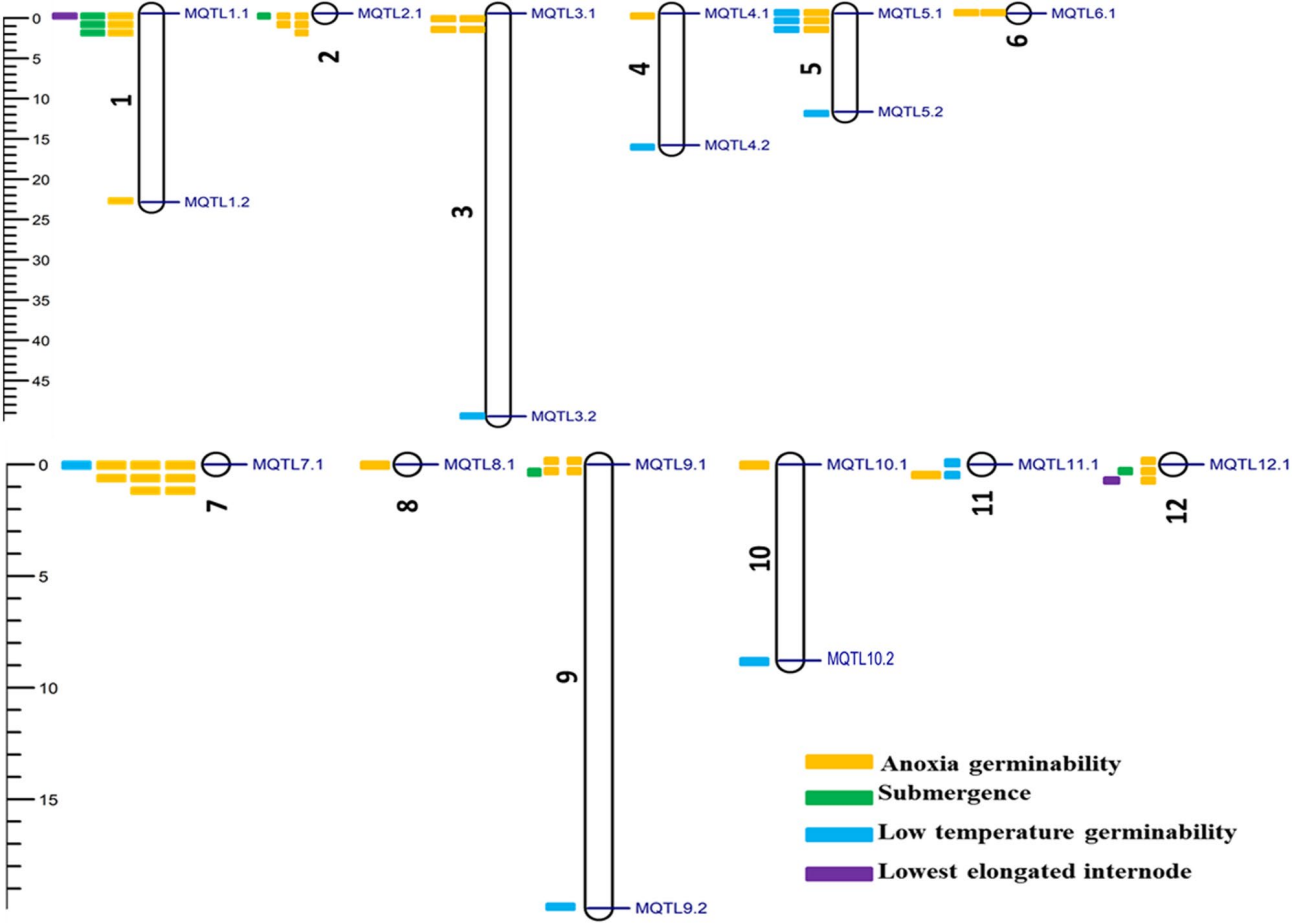
The chromosome distribution of MQTL for submergence tolerance

For consensus QTL prediction, the LOD score, PVE, and chromosome position were considered. As a consequence, a total of 103 QTL out of 120 QTL were successfully projected on the consensus map (Fig. 3a; Supplementary Table 1). The remaining 17 QTL were not projected due to lack of common markers between initial QTL and the reference map and low PVE scores resulting in large confidence intervals. Moreover, there was a high correlation between initial and projected QTL on rice chromosomes with pearson correlation coefficient (r) of 0.93 (Fig. 3b). For the projected QTL, 50 QTL were identified as major QTL (PVE ≥ 10%), while 53 were minor QTL (PVE < 10%). The QTL meta-analysis confined the initial QTL to 18 MQTL, which contained only 56 of the projected QTL (Fig. 4; Supplementary Table 2). Of the identified MQTL, 50% (9/18) were composed of two or more initial QTL and 50% (9/18) constituted only single initial QTL. The MQTL with relatively high initial QTL harbored QTL from diverse bi-parental populations indicating that they are more reliable and stable for submergence tolerance improvement in rice. Notably, chromosomes 1, 2, 3, 5, 7, 9, and 12 had MQTL with high initial QTL ranging from nine QTL (MQTL7.1) to four QTL (MQTL3.1).

The CI of MQTL ranged from 0.02 to 40.7 cM with an average of 7.34 cM, while the CI of projected QTL ranged from 0.88 to 65.22 cM with an average of 25.22 cM. There was a significant difference between the mean CI of MQTL and the initial QTL that mapped on the rice chromosomes (Fig. 3c). Also, MQTL exhibited significant fold reduction in CI across rice chromosomes ranging from 0.12 for MQTL on chromosomes 2 and 7 to 20.37 cM for MQTL on chromosome 3. The PVE scores of individual MQTL ranged from 0.15 to 31.23% with a mean score of 11.85% (Supplementary Table 2). Per the criteria established by Löffler et al. [49], MQTL to be considered for marker-assisted breeding should possess a high number of initial QTL, high PVE values of initial QTL, and relatively small CI. The following criteria were then employed to select the most important MQTL: (i) MQTL involving at least four initial QTL, (ii) mean PVE > 10%, and (iii) CI < 2 cM. Based on this, four MQTL were selected to be the most significant MQTL and thus named as core MQTL. These four MQTL were as follows: MQTL2.1, MQTL3.1, MQTL7.1, and MQTL12.1 (Table 2).

**Table 2:** List of core MQTL identified in this study

MQTL ID^a^	Position (cM)	CI (cM)^b^	Genetic interval (cM)	Initial QTL	PVE (%)^c^	Flanking markers
MQTL2.1	0.25	0.12	0.14–0.26	6	12.18	RM12578 - RM12381
MQTL3.1	0.00	0.02	0.0–0.02	4	18.54	GNMS1289 - RM3265
MQTL7.1	0.00	0.12	0.11–0.23	9	12.01	S2329 - HV7-02
MQTL12.1	0.41	0.29	0.28–0.51	5	11.46	HV12-11 - TEL2A

^a^Meta-QTL ^b^Confidence interval ^c^Mean phenotypic variance explained

### Validating MQTL by GWAS-MTAS

**Fig. 5:**
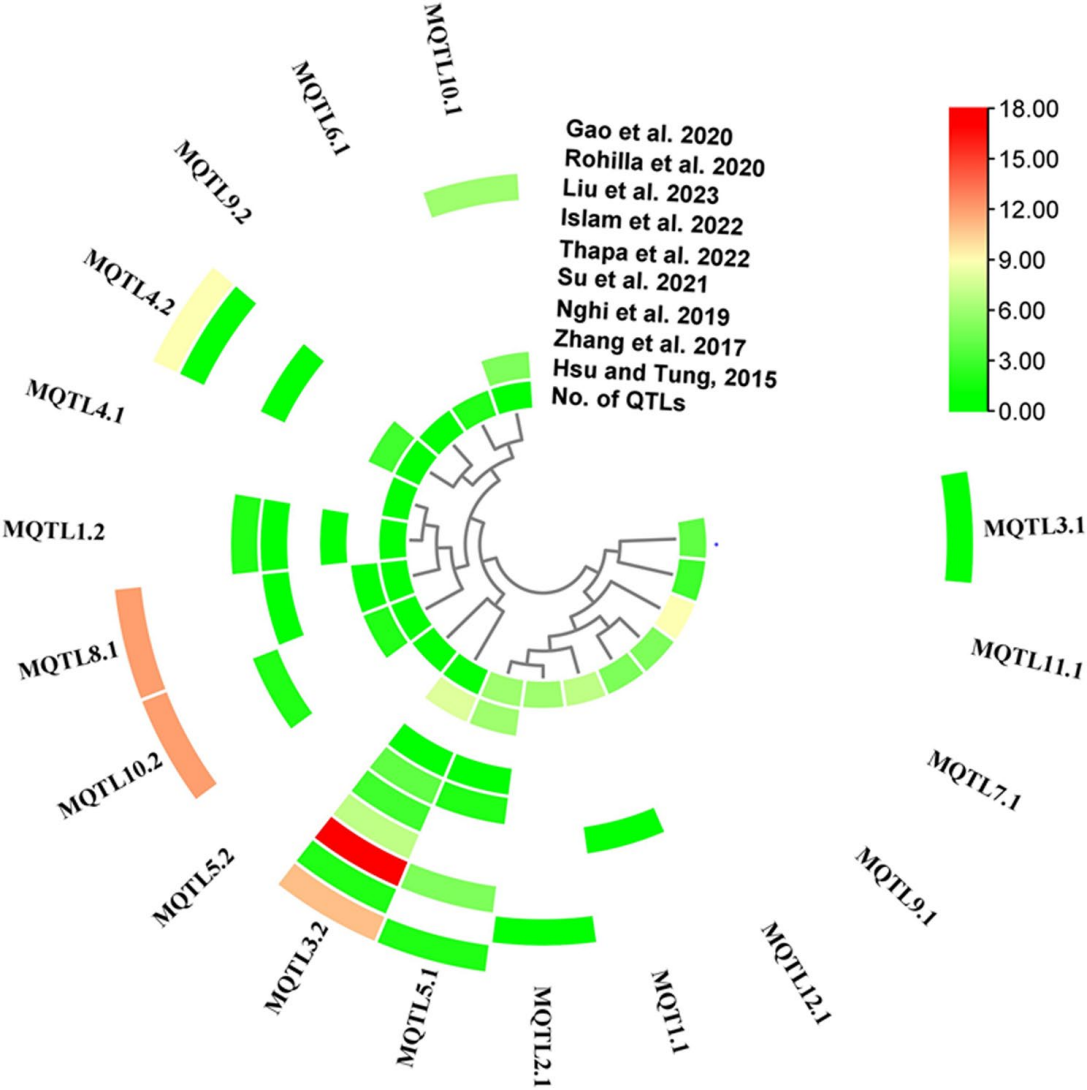
Validation of MQTL by marker-trait associations (MTAs) on rice submergence-related traits from GWAS with nine diverse natural populations. Color codes showed the number of MTA overlapping the identified MQTL

**Fig. 6:**
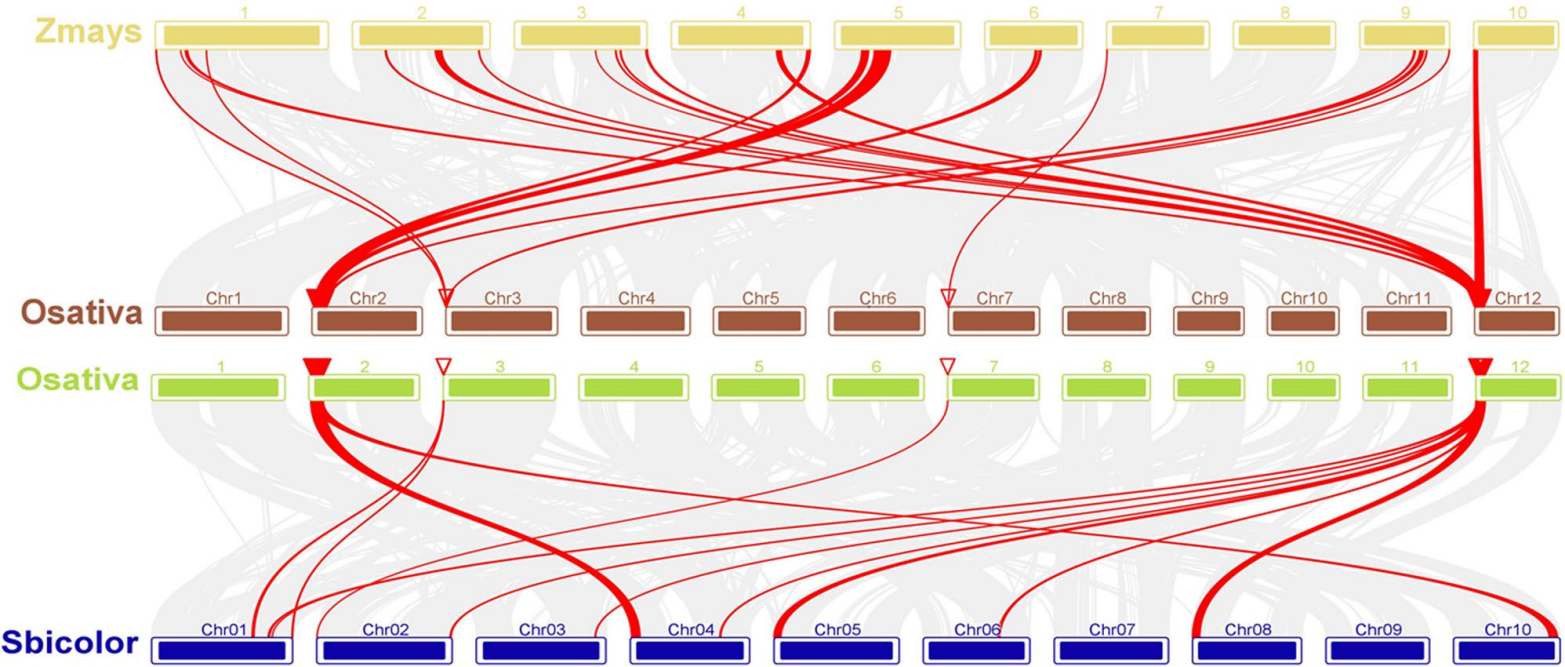
Syntenic regions among rice, sorghum, and maize genomes. The gray lines in the background depict collinear blocks within pairs of rice and each other species; red lines show syntenic gene pairs

To confirm the reliability of the meta-analysis, MQTL were verified from GWAS studies involving submergence-related traits published in recent years (Supplementary Table 3). Intriguingly, 55.56% (10/18) of MQTL in this study co-localized with 124 SNP signals associated with submergence-related traits (Fig. 5). The number of MTA overlapping the identified MQTL also varied from one to 17 in nine association studies. Each of the 10 MQTL overlapped at least one MTA. In particular, MQTL3.2, MQTL5.1, MQTL4.2, MQTL8.1, MQTL10.2, and MQTL10.1 overlapped 53, 16, 14, and 11 MTA. In light of the synteny among rice, maize, and sorghum, the corresponding CGs identified in the core MQTL regions were examined on the genomes of sorghum and maize. As expected, the core MQTL loci were conserved among rice relatives indicating that comparative genomics analysis could be useful in identifying candidate genes and transferring information across cereal crops (Fig. 6).

### Mining of candidate genes underlying core MQTL regions and exploration of their functional classification

**Fig. 7:**
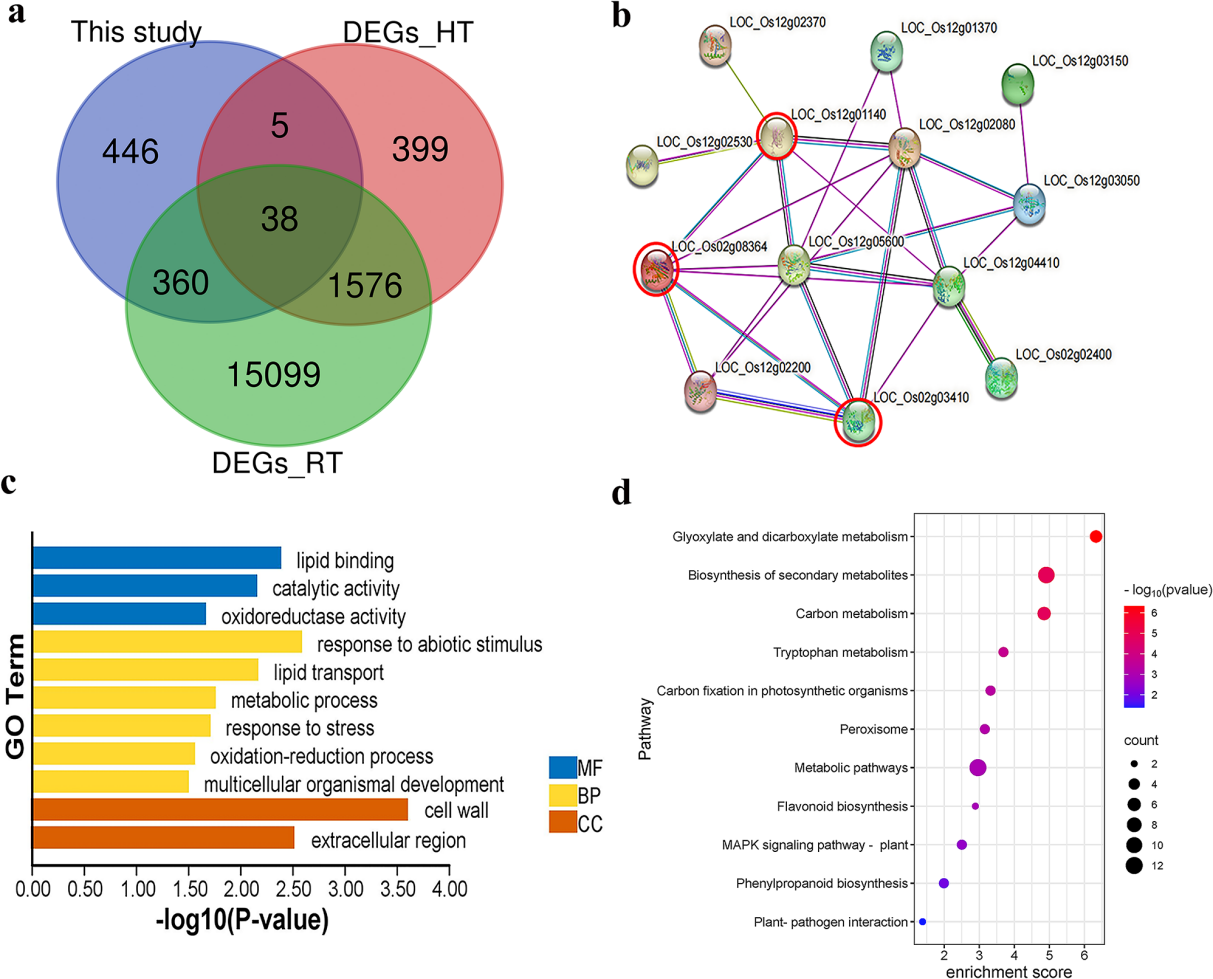
Analysis of transcriptomic datasets and genes in the core MQTL regions. **a** Venn diagram showing the number of differentially expressed genes (DEGs) between transcriptome data and genes in the core MQTL loci, **b** Protein-protein interaction among the common DECGs, **c** GO terms for DECGs in core MQTL regions, **d** KEGG enrichment pathways for DECGs in core MQTL regions

A total of 858 putative CGs were mined in the core MQTL regions; these candidate genes included 28 genes with protein kinase domain, 22 zinc finger family proteins, 17 cytochrome P450 proteins, 11 DUF domain proteins, 7 WRKY, and four glycosyltransferase domain-containing proteins (Fig. 3d; Supplementary Table 4). To validate CGs underlying core MQTL regions, submergence-responsive genes were obtained from two independent transcriptome studies. Based on DEGs datasets, 5,124 and 25,614 genes were differentially expressed in response to anaerobic germination during flooding. Overall, 38 common DEGs were identified between DEGs studies and genes found in the core MQTL regions, hence they were designated as DECGs (Fig. 7a; Supplementary Table 5). Next, a protein-protein interaction (PPI) network was constructed to predict the relationship among DECGs. The PPI analysis revealed three main hub gene interactions; *LOC_Os02g03410*, *LOC_Os02g08364*, and *LOC_Os12g01140* interacted with five genes involved in calcium flux signaling, reactive oxygen species (ROS) detoxification, and cellular stress signaling (Fig. 7b; Supplementary Table 6).

Ultimately, GO and KEGG pathway enrichment of DECGs were explored to decipher genes’ functional classification. As expected, three main GO terms were revealed including biological processes (six subfunctions), molecular functions (three subfunctions), and cellular components (two subfunctions) (Fig. 7c). For biological processes, the most enriched GO terms involved response to abiotic stimulus, lipid transport, metabolic process, and stress response. For molecular functions, the DECGs were mainly enriched in lipid binding, catalytic activity, and oxidoreductase activity, while the main enriched terms for cellular components involved cell wall and extracellular region. The KEGG pathway enrichment analysis showed several mechanisms involved in submergence tolerance, among them include glyoxylate and dicarboxylate metabolism, biosynthesis of secondary metabolite, and carbon metabolism pathways (Fig. 7d; Supplementary Table 7).

### *In silico* expression analysis of DECGs and cis‑acting regulatory elements in the promoters of submergence-responsive genes

**Fig. 8:**
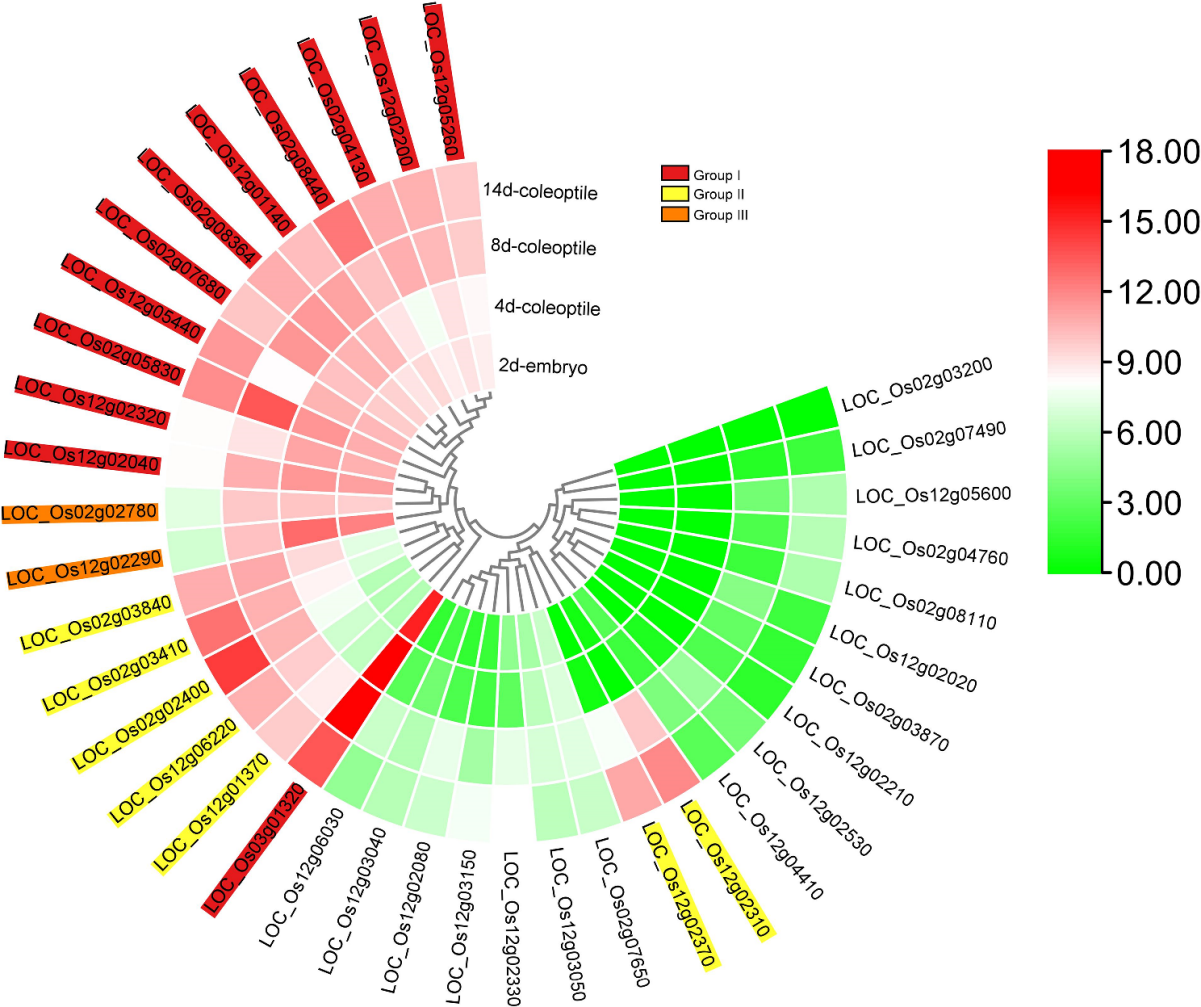
Expression profiles of 38 differentially expressed candidate genes (DECGs) in two different tissues under submergence conditions. Transcriptome data were downloaded from NCBI

To pinpoint potential DECGs involved in submergence regulation, *in silico* expression analysis was performed. The expression patterns of these genes were assessed during developmental stages under submerged conditions. Of the 38 DECGs evaluated, only 21 genes were highly upregulated in embryo and coleoptile at 2, 4, 8, and 14 days, in response to flooding/submergence (Fig. 8). Based on their expression profiles, these putative genes could be divided into three groups (Fig. 8). The group I genes were highly expressed across all four stages (2 days in embryo, while others were expressed in coleoptile at 4, 8, and 14 days). In group II, genes were specifically highly expressed in coleoptile at 8 and 14 days, while group III genes were highly upregulated in embryo and coleoptile at 2, 4, and 8 days submergence. Therefore, we hypothesized that these genes could be promising candidate genes modulating rice submergence tolerance.

**Fig. 9:**
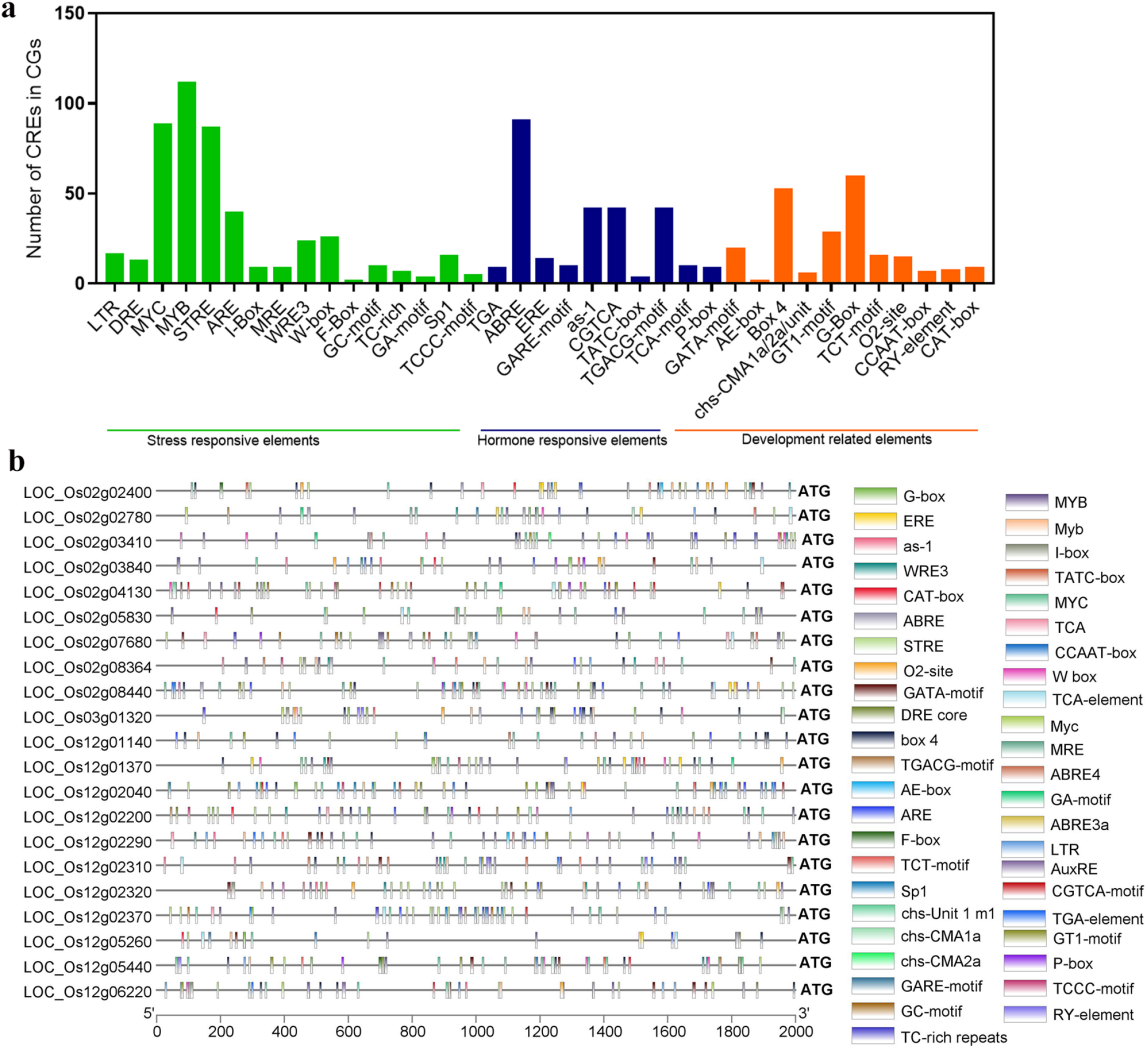
Analysis of cis-regulatory elements (CREs) in promoter regions of 21 potential DECGs identified in this study

*Cis*-acting regulatory elements (CAREs) in gene promoters play crucial roles in gene expression regulation [50], therefore it is imperative to study CAREs located in the promoter regions of the putative DECGs identified in this study. Within the 2 kb promoter regions of the 21 potential DECGs, a total of 977 CAREs were detected; these CAREs were further divided into three groups, thus stress-responsive elements, hormone-responsive elements, and development-related elements (Fig. 9; Supplementary Table 8). Among the stress-responsive elements include those involved in anaerobic induction (ARE), stress (As-1, TC-rich repeats, STRE), and low temperature (LTR). Many CAREs, such as abscisic acid (ABRE), ethylene (ERE), Me-JA (TGACG-motif), salicylic acid (TCA-motif), gibberellin (TATC-box, GARE-motif) and auxin (TGA) are among phytohormone responsiveness elements. Interestingly ABRE was the most abundant element located in the promoter regions of 21 DECGs. Also, shoot and root meristem expression (CAT-box), light responsiveness (G-box, TCT-motif, etc.), and zein metabolism-mediated *cis*-elements were all involved in the development processes. These results suggest that a complex regulatory network modulates the transcription of genes involved in submergence tolerance.

### Haplotypes of putative DECGs

**Fig. 10:**
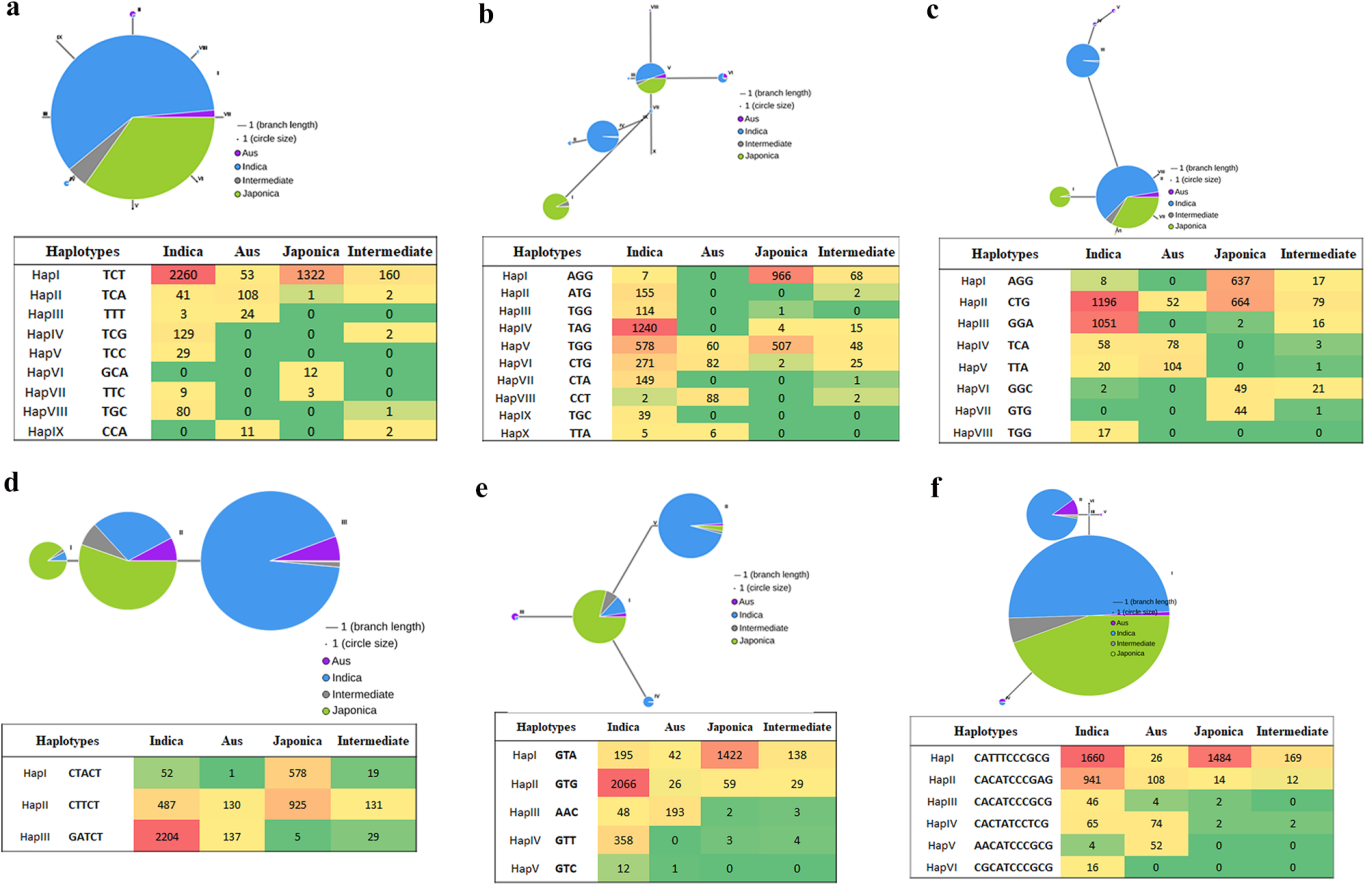
Haplotype analysis of five potential DECGs. Circle size corresponds to the number of samples for a given haplotype. Lines between haplotypes indicate mutational steps between alleles. The tables give a summary of the number of haplotypes present in individual genes and their distribution in rice subpopulations. Different colors show the number and or the density of accessions corresponding to subpopulations

Rice genes associated with abiotic stress tolerance have been reported to exhibit a specific distribution of mutation between *indica* and *japonica* groups [51, 52]. Based on this, we investigated the haplotype distribution in the 21 putative DECGs. The results revealed that the 21 DECGs contributed a total of 188 haplotypes (Fig. 10; Supplementary Fig. 2). It was found that the haplotypes in individual genes differentiated significantly in *indica*, *japonica*, aus, and intermediate accessions with the majority of haplotypes distributed in indica rice. Haplotypes in these genes ranged from 20 in *LOC_Os12g02370* to three in *LOC_Os03g01320*. The candidate genes in this study showed a great deal of genetic differentiation and genetic distance mainly between *indica* and *japonica* accessions.

### Validation of meta-QTL

**Fig. 11:**
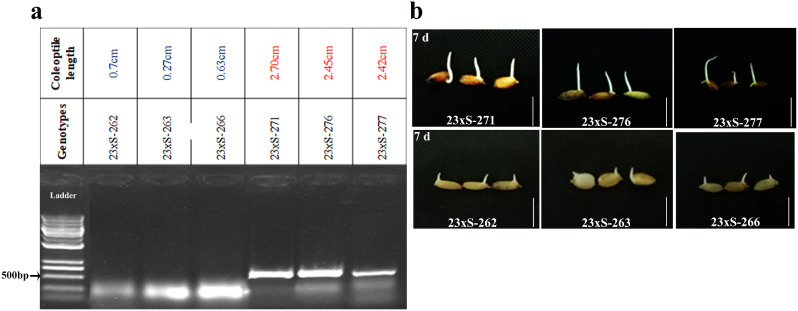
Validation of marker S2329. **a** Validation of marker S2329, peak marker of MQTL7.1 on a set of three long coleoptile length and three short coleoptile length genotypes under submergence condition. The allele with 250 bp related to short coleoptile length and 500 bp allele associated with long coleoptile length, values represent the mean of coleoptile length of three replicates, **b** Representative images of genotypes subjected to submergence stress for 7 days. Scale bars denote 10 mm

Based on the consensus map, one of the peak markers flanking the core MQTL was selected for MQTL validation. Marker S2329 from MQTL7.1 was used for MQTL validation. Interestingly, S2329 could differentiate the genotypes into two phenotypic groups, i.e., short coleoptile length (sensitive lines) and long coleoptile length (tolerant lines) when genotypes when submerged. The mean coleoptile length of sensitive lines ranged from 0.27 to 0.7 cm while the mean coleoptile length of tolerant lines ranged from 2.42 to 2.70 cm (Fig. 11a and b). The allele with an amplicon size of 500 bp was found specifically related to tolerant genotypes (long coleoptile length) while an amplicon size of 250 bp was related to sensitive genotypes (short coleoptile length) (Fig. 11a; Supplementary Fig. 3).

## Discussion

Due to rising costs, labour shortages, and water shortages, farmers are now practicing direct seeding in the puddled field in rainwater and irrigation systems across the globe [53, 54]. In such a case, the ability of rice to survive under submergence/flooding conditions during germination is critical. Although rice seeds can germinate under hypoxic or even anaerobic conditions and extend the coleoptile, anaerobic conditions are extremely detrimental to seedling growth and establishment at the early seedling stage [11, 22, 55]. Numerous submergence-related QTL have been identified in the past few decades thanks to the discovery of molecular markers and breakthroughs in QTL mapping strategies [20, 21]. However, the use of these QTL in breeding programs faces major bottlenecks due, in part, to low PVE values and large confidence intervals.

In the present study, we conducted a meta-analysis of 120 previously reported QTL underlying submergence-related traits from 16 independent studies and discovered that these 120 QTL were unevenly spread across the rice chromosomes, with a large number of QTL located on chromosomes 7, 1, and 5 and the least QTL on chromosomes 4, 8, and 11 (Fig. 1) which agreed with our previous study [39] and studies conducted by Wu et al. [56] and Khahani et al. [37]. One of the interesting findings of this study is that the PVE by most of the initial QTL was low indicating that minor effect loci are at play in influencing rice submergence tolerance. The CI of 18 MQTL identified in this study was 3.44 times lower than that of the initial QTL studies indicating a possible close linkage of associated markers to the MQTL, thus enabling flexibility of introgressing MQTL in marker-assisted breeding. This finding is consistent with our previous report [39] and other studies in rice and wheat [30, 57, 58]. However, for individual MQTL, we found that MQTL3.2 had a large confidence interval (40.7 cM) although did not exceed the CI of the initial QTL (Supplementary Table 2). This phenomenon could be ascribed to low initial QTL involvement and small CI associated with the reported QTL [59]. Moreover, Saini et al. [34] observed a similar event occurring in their meta-analysis study involving wheat and suggested a possible occurrence of low recombination frequency in the MQTL locus. Furthermore, the distribution of MQTL was uneven across the rice chromosomes with chromosomes 1, 3, 4, 5, 9, and 10 constituting the highest number of MQTL than other chromosomes with a single MQTL.

To confirm the reliability of our MQTL, we validated MQTL results with markers reported in GWAS and found that more than 50% of the 18 MQTL overlapped with loci from those studies, which corroborated with the previous report [60–62]. The MQTL colocalized with loci at least twice across independent GWAS, of which MQTL3.2 and MQTL5.1 were detected in eight and five GWAS reports, respectively (Fig. 5). This outcome indicated the reliability of our MQTL. In addition to marker-assisted selection (MAS), MQTL overlapping markers from GWAS can also be used to clone genes involved in MQTL with a higher degree of confidence.

A set of four MQTL were designated as core MQTL. Each of these MQTL was characterized by relatively small CI (CI < 2 cM), high PVE (> 10%), and involvement of at least four initial QTL. Interestingly, all four core MQTL contained initial QTL from diverse genetic backgrounds and different environments [32, 63]. By synteny analysis, we showed that these core MQTL were conserved between rice, maize, and sorghum genomes (Fig. 6) [64]. These syntenic regions constituted numerous unexploited genes in rice which can be targeted for functional validation in future research. The synteny analysis also provides the opportunity to comprehend the molecular and evolutionary basis of submergence tolerance across cereal crops. Moreover, gene-based functional marker development in these regions can be feasible in breeding for submergence tolerance in cereals.

The candidate genes present in MQTL are prioritized for allele mining [65]. In the selected core MQTL regions, we mined 858 gene models including 173 genes with unknown functions. Some of the well-known proteins encoded genes associated with submergence stress included aldehyde dehydrogenase, F-box domain proteins, WRKYs, cytochrome P450, glycosyltransferase, hypoxia-induced protein, PP2C, homeobox domain protein, sucrose synthase, protein kinase, zinc fingers, amino acid permease family, ethylene-responsive factors/AP2 domain, and DEAD-box ATP dependent proteins (Fig. 3). In previous studies, aldehyde dehydrogenase is involved in rice submergence tolerance and related abiotic stresses [66–68]. The expression of aldehyde gene (*Aldh2a*) in rice was induced in seedlings under flooding [66]. In a recent study, a gene encoding glucosyltransferase enhanced submergence tolerance in rice during germination [8]. *OsUGT75A* promoted coleoptile elongation by limiting abscisic acid and jasmonic acid levels. A well-known *SUB1A* gene confers tolerance to rice submergence; *SUB1A* encoding ethylene response factor-like protein binds to the promoter of MAPK3 (Mitogen-activated protein kinase3) and controls its expression during submergence stress signaling [69]. In rice coleoptiles, a considerable number of ERFs including *ERF60*, *ERF67*, and *ERF68* genes have been implicated under anaerobic conditions and also belong to the ERF subgroup VII [70]. Overall, these findings substantiate that the core MQTL regions embodied potential candidate genes involved in response to submergence and can be utilized for map-based cloning of submergence tolerance genes.

To enable robust identification of candidate genes associated with submergence, we validated genes located in the core MQTL regions by comparing them with two publicly available transcriptome datasets and found that only 38 genes (DECGs) overlapped. PPI is a useful method for understanding how genes function at the cellular level [71–73]. In the present study, we found three main hub-gene interactions among DECGs. These genes (*LOC_Os02g03410*, *LOC_Os02g08364*, and *LOC_Os12g01140*) interacted with genes involved in ROS detoxification, calcium flux signaling, and cellular stress signaling (Fig. 7), indicating that complex regulatory network of genes influenced rice seed germination under submerged conditions [74, 75]. The analysis of GO and KEGG pathway enrichment of DECGs in core MQTL regions is vital for understanding mechanisms underlying submergence tolerance in rice. Most of the genes in the core MQTL regions were significantly enriched in cell wall, abiotic stimulus, extracellular region, lipid binding, lipid transport, and catalytic activity [75]. Interestingly, we found that genes in these regions were enriched in pathways including carbon metabolism, MAPK signaling pathway-plant, tryptophan metabolism, and isoflavonoid biosynthesis pathway which have been reported to play important roles in flooding tolerance [71]. Also, pathways such as glyoxylate and dicarboxylate metabolism, biosynthesis of secondary metabolites, metabolic pathways, carbon fixation in photosynthetic organisms, and phenylpropanoid biosynthesis might be involved in submergence tolerance. To pinpoint potential DECGs, we used expression data generated under rice submergence conditions during germination deposited in NCBI database. Accordingly, we found a total of 21 genes with high and stage-specific expression profiles in embryo at two days, and coleoptile at four,, eight, and 14 days (Fig. 8). These DECGs exhibited tissue and development-dependent expression profiles and are considered potential CGs directly involved in submergence tolerance (Table 3). For instance, *LOC_Os02g02780* encoding protein kinase was significantly upregulated in embryo and coleoptile. *LOC_Os02g07680* and *LOC_Os12g05440*, encoding cytochrome P450 were highly expressed in embryo and coleoptile at all stages of development under anaerobic germination. *LOC_Os12g02040* encoding expressed protein was highly induced in embryo and during coleoptile elongation under anaerobic conditions (Fig. 8). A total of 11 reported loci were found in MQTL2.1, MQTL3.1, and MQTL12.1 regions (Table 3). OsCatA encodes catalase isozyme A enhanced drought tolerance by modulating ROS homeostasis associated with root growth and photorespiration [76]. The orthologue of *OsCatA* in maize *Zm00001d014848* plays an essential role in ROS scavenging by converting H_2_O_2_ into H_2_O and O_2_ [77, 78]. The locus *OsSGL* and its orthologue *ZmDUF1645* in maize increased grain yield and stress tolerance by regulating the expression of genes involved in the cytokinin signaling pathway [79–81]. The gene *OsWRKY71* (*LOC_Os02g08440*) was reported to play a positive role in cold [82] while its orthologue ZmWRKY40 (Zm00001d015515) in maize was specifically up-regulated in hydrotropic maize root [83]. Similarly, salt stress and drought stress responses in rice are positively regulated by locus *OsCPK4* (*LOC_Os02g03410*) through the protection of cellular membranes from oxidative damage [84]. Although none of the reported genes within MQTL2.1 were found directly involved in submergence stress tolerance, their roles in biological processes associated with abiotic stress response and yield proved significant, hence warranting further studies using submergence stress as a case study. In the MQTL3.1 region, *qLTG3-1* (*LOC_Os03g01320*) has been proven to modulate rice seed germination under low-temperature conditions [85]. Moreover, the findings of previous studies indicated that the clone genes found in MQT12.1 played diverse roles in plant stress response [86]. For instance, a high expression of *CYP94C2b* (*LOC_Os12g05440*) encodes cytochrome P450 inactivated jasmonic acid biosynthesis thereby enhancing salt tolerance in rice [86]. The identified candidate genes in these MQTL regions provide clues for their possible roles in submergence tolerance with similar evolutionary trajectories and conserved functions between cereal crops. Breeders can use these results to track down candidate genes and employ marker-assisted selection in breeding programs of cereals under submergence stress.

**Table 3:** List of potential candidate genes identified in this study

MQTL ID	Candidate genes	Functional description	Gene symbol
MQTL2.1	*LOC_Os02g02400*	catalase isozyme A	*OsCatA*
	*LOC_Os02g02780*	protein kinase family protein	*ACTPK1*
	*LOC_Os02g03840*	expressed protein	
	*LOC_Os02g04130*	DUF1645 domain-containing protein	*OsSGL*
	*LOC_Os02g05830*	ribulose bisphosphate carboxylase small chain, chloroplast precursor	*OsRBCS1*
	*LOC_Os02g07680*	cytochrome P450	
	*LOC_Os02g08364*	protein phosphatase 2 C	
	*LOC_Os02g08440*	WRKY71, expressed	*OsWRKY71*
	*LOC_Os02g03410*	calcium/calmodulin-dependent protein kinases	*OsCPK4*
MQTL3.1	*LOC_Os03g01320*	LTPL116 - Protease inhibitor/seed storage	*qLTG3-1*
MQTL12.1	*LOC_Os12g01370*	fatty acid desaturase, putative, expressed	*OsFAD3*
	*LOC_Os12g02040*	hypoxia-responsive family protein	
	*LOC_Os12g02200*	calcium/calmodulin depedent protein kinases	*OsCIPK14*
	*LOC_Os12g02290*	LTPL23 - Protease inhibitor/seed storage	
	*LOC_Os12g02310*	LTPL11 - Protease inhibitor/seed storage	
	*LOC_Os12g02320*	LTPL12 - Protease inhibitor/seed storage	
	*LOC_Os12g02370*	chalcone–flavonone isomerase	*OsCHIL2*
	*LOC_Os12g05260*	phytosulfokines precursor	
	*LOC_Os12g05440*	cytochrome P450	*CYP94C2b*
	*LOC_Os12g06220*	harpin-induced protein 1 domain-containing protein	
	*LOC_Os12g01140*	ACG kinases include homologs to PKA, PKG and PKC	

CAREs are important DNA sequences located in the promoter region of genes that allow the binding of transcription factors leading to either enhancing or silencing gene expression when unfavorable conditions prevail [48]. In our study, all 21 submergence-associated genes (DECGs) contained three distinct functional categories of cis-elements such as stress-responsiveness, phytohormone responsiveness, and development-related elements (Fig. 9). For instance, ARE and GC-motif located in the promoter region of *OsWRKY62* upregulated transcription in response to oxygen deficiency. W-box, a stress-responsive element was found in the promoter of *OsWRKY11*, *OsWRKY56*, and *OsWRKY62* and upregulated their expression under submergence stress [87, 88]. Surprisingly, phytohormone-responsive elements ABRE occurred more frequently in 21 DECGs which echoed its importance in submergence regulation (Fig. 9). Stress-responsive *cis*-elements DRE as well as phytohormone-responsive elements including ABRE have all been implicated in response to submergence [89].

Haplotype strategy which utilizes genome sequence information and phenotype data has proven useful in excavating natural allelic variants present in different genotypes to develop tailored-made superior cultivars [90, 91]. By analyzing the genetic diversity of 21 DECGs, we found that the haplotypes of these genes significantly differentiated mainly in *indica* and *japonica* subspecies [31]. The differences in tolerance levels between *indica* and *japonica* rice are probably due to these haplotypes, which have important implications for breeding rice varieties suitable for direct seeding under flooding conditions. However, this requires further validation by exploring genetic variants in ideal rice cultivars to substantiate gene function.

Among the core MQTL identified in this study, we selected MQTL7.1 for validation on extreme genotypes including three sensitive lines (short coleoptile length) and three tolerant lines (long coleoptile length). Consistently, marker S2329 associated with MQTL7.1 successfully differentiated two extreme genotypes (Fig. 11). Segregation of marker amplicons among the extreme genotypes validates their close linkage with MQTL7.1. S2329 was identified as the peak marker of MQTL7.1, suggesting its close linkage with MQTL7.1. It is worth noting that the validated marker has the potential to be used in marker-assisted selection by integrating MQTL7.1 region into an elite background to improve submergence tolerance in rice.

### Concluding remarks

In the present study, we integrated QTL conferring submergence tolerance in rice leading to the identification of 18 MQTL. More than 50% of the identified MQTL were validated by GWAS signals. Four MQTL were designated as core MQTL because they contained a high number of initial QTL and are potential regions for excavating submergence-associated candidate genes. Integrated DEGs from public transcriptome datasets and genes in the core MQTL regions revealed 38 genes as DECGs. Further expression analysis of 38 DECGs showed significant upregulation of 21 DECGs in various rice organs under submergence conditions. Some putative genes encoding cytochrome P450, WRKY, hypoxia-responsive protein, calcium/calmodulin-dependent protein kinases, zinc finger protein, and protein phosphatase 2 C were located in the core MQTL regions. The 21 DECGs showed a great deal of genetic differentiation and genetic distance mainly between *indica* and *japonica* accessions. The meta-QTL and candidate genes identified in this study provide valuable information for MAS and future functional validation experiments by CRISPR/Cas9 and other techniques aimed at breeding of rice varieties suitable for direct seeding and can help decipher the molecular mechanisms underlying submergence tolerance during seed germination.

### Electronic supplementary material

Below is the link to the electronic supplementary material.

Supplementary Material 1

Supplementary Material 2

Supplementary Material 3

## Data Availability

All data supporting the conclusions of this article are provided within the article and its supplementary.
