# Supplementary material for: Integrated meta-analysis and transcriptomics pinpoint genomic loci and novel candidate genes associated with submergence tolerance in rice
Source: BMC Genomics. 2024 Apr 4;25:338. doi: 10.1186/s12864-024-10219-z (PMC10993490; doi:10.1186/s12864-024-10219-z)
Supplement: Supplementary file 2 — Supplementary Material 2 [file 12864_2024_10219_MOESM2_ESM.pdf]

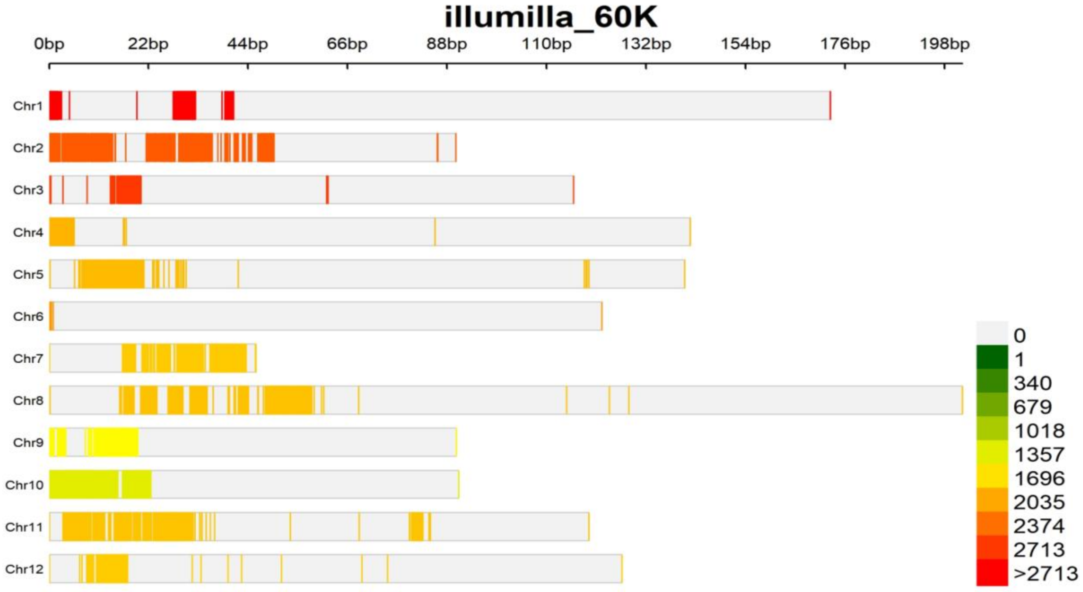

Supplementary Fig. 1 Distribution of markers on the consensus map used for meta-analysis of QTL. Different colours indicate the density of markers on each chromosome.

a

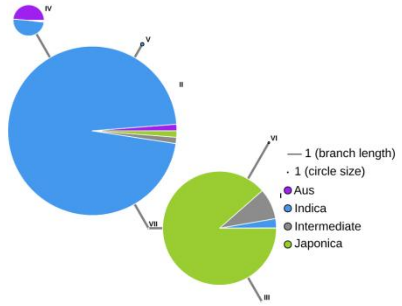

Haplotype		Indica	Aus	Japonica	Intermediate
HapI	GCGAATTA	43	2	1476	146
HapII	GCAAACTG	2386	31	30	30
HapIII	TCGGATTA	14	3	0	1
HapIV	GCGACCTG	224	222	4	5
HapV	GTAAACTG	48	0	0	0
HapVI	GTGAACCA	25	0	0	0
HapVII	GCAAATTA	1	10	1	1

b

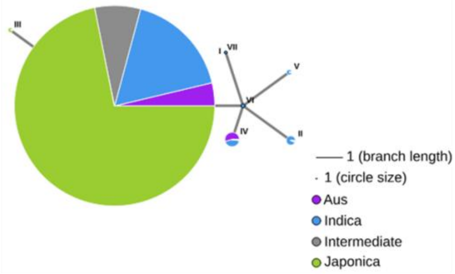

Haplotype		Indica	Aus	Japonica	Intermediate
HapI	GAGGG	262	57	1098	113
HapII	GGGAG	65	0	2	2
HapIII	GGGAT	2	0	30	2
HapIV	GGGGC	47	60	1	1
HapV	GGGAA	38	1	0	0
HapVI	AGAAG	32	0	0	0
HapVII	GGGTG	23	0	0	0

c

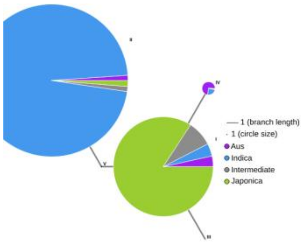

Haplotype		Indica	Aus	Japonica	Intermediate
HapI	AGGGGCGCG	72	61	1475	145
HapII	AGGAACACG	2592	30	33	29
HapIII	GGAGGGGCG	13	4	0	1
HapIV	ACGGGCGTT	54	167	2	5
HapV	AGGGGCACG	17	0	0	0

d

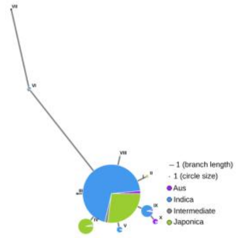

Haplotype		Indica	Aus	Japonica	Intermediate
HapI	TCACGCCTGTTGTACGTATAAATTGATACTTTCCTGCAACAGGTTCCG	1755	42	680	43
HapII	TCACGCCTGTTGTACGTATAAGTTGATACTTCCCTGCAACAGGTTCCG	2	0	125	0
HapIII	TCACGCCTGTTGTACATATAAATTGATACTTTCCTGCAACAGGTTCCG	73	0	0	0
HapIV	TCACGCCTGTTGTACGTATAAATTGATACTTTCCTGCAACAAGTTCCG	11	2	639	17
HapV	TCACGCTTGTTGTACGTATAAATTGATACTTTCCTGCAACAGGTTCCG	245	0	3	4
HapVI	TCCCACCAACCCAGTGCACGGAGACTGGGTCTCCTGCAACAGGTTCCG	81	1	6	99
HapVII	TCCCAACAACCCAGTGCGCGGAGACTGGGTCTAACAGTGGTGAACTGA	57	0	0	0
HapVIII	TCACGCCTGTTGTACGTATAAATTGATACACTCCTGCAACAGGTTCCG	0	1	40	2
HapIX	GCACGCCTGTTGTACGTATAAATTGATACTTTCCTGCAACAGGTTCCG	482	28	0	11
HapX	GAACGCCTGTTGTACGTATAAATTGATNCTTTCCTGCAACAGGTTCCG	36	192	6	3

e

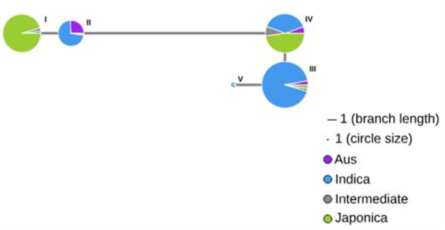

Haplotype		Indica	Aus	Japonica	Intermediate
HapI	AGCGGTATGTATAAAACCTACTGCATGGTGCCTTCTGGACTCCGCAGCCTGCACG	19	0	839	24
HapII	AGCGGTATGTATAAAACCTACTGCATGGCGCCTTCTGGACTCCCCAGCCTGCACG	426	155	6	7
HapIII	NNNNNNNCNNNNNNNNNNNNNNNNGTCGGTACCTCAGCCGCTTGCGTATGCCACAG	992	31	27	20
HapIV	NNNNNNNCNNNNNNNANNNNNNNNGTCGNNACCTNAGCCGCTTGCGTATGCNNCAC	351	51	429	72
HapV	NNNNNNNCNNNNNNNANNNNNNNNGTCGNNANNGNAGGCGATTGCGTATGCCACAG	110	0	1	1

f

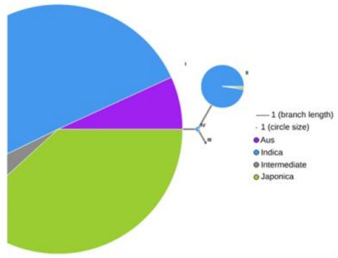

Haplotype		Indica	Aus	Japonica	Intermediate
HapI	CGCG	1979	269	1504	178
HapII	GTCA	665	0	8	7
HapIII	CGTA	23	0	0	0
HapIV	CGCA	75	0	0	1

g

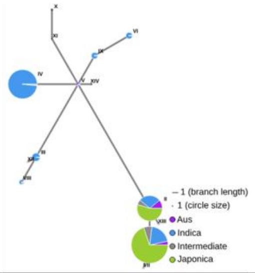

	Haplotypes	Indica	Aus	Japonica	Intermediate
HapI	CTTTAATGCTACATATTGTGGTTCTACAGTTTTCGGGGAGGAGAAATCATCTGTCCGCCATAAGGGGTTTTT	260	44	913	95
HapII	CTTTAATGCTACATATTGTGGTTCTACAGTTTTCGGGGAGGAGAAATCATCTGTCCGCCATAAGGGGTTCTT	254	100	493	58
HapIII	CCTTCCTGCTACATATTAGTGTTTTACAGCTCACAGAAGGGCGGACACGCCTGTCAGCGGCAAGGGGCNNTT	287	0	0	2
HapIV	CCTTCATGCTACATATTATTGTTTTTCAGCGCTCGGGGGAGCAAACACGCTTGTTNTCCACAGGAAGCNNTT	1025	0	4	11
HapV	CCTTCATGCTACATATTATTGTTTTTCAGCTCTCGAGGGGGCAAACACGCTTGTTNTCGACAAGGGGCCCTT	36	94	0	3
HapVI	CCCTCATGCTACATACAATTGTTTTTCAGTTCTCGGGGGGGCAACCACGCTCGTTNTCGACAAGGGGCCCTT	228	0	0	1
HapVII	CTTTAATGCTACATATTGTGGTTCTACAGTTTTCGGGGAGGAGAAATCATCTGTCCGCCATAAGGGATTNTT	3	0	9	0
HapVIII	CCTTCATGCTCCACATTAGTGTTTTACAGCTCACAGAAGGGCGGACACGCCTGTCNGCGACAAGGGGCCNTT	133	8	34	0
HapIX	CCTTCATGCTACATGCTATTGTTTTTCAGTTCTTGAGGGGGCAACCACGCTTGTTNTCGACAAGGGGCCCTT	223	0	1	2
HapX	CCTTCACTCTACATATTATTATCTTTTGACTCTCGGGGGGGCAGACATGCTTGTTNTCCACAAGGAGCCCTT	60	0	0	0
HapXI	CCTTCATTCTACATATTATTATCTTTTAACTCTCGGGGGGGCAGACACGCTTGTTNTCCACGAGGGGCCCTT	14	0	0	0
HapXII	CCTTCCTGCTACATATTAGTGTTTTACAGCTCACAGAAGGGCGGACACGCCTGTCAGCGGCAAAGGGCCCTT	10	0	1	0
HapXIII	CTTTAATGCTACATATTGTGGTTCTACAGTTTTCGGGGAGGAGAAATCATCTGTCCGTCATAAGGGGTTCTT	1	0	14	0
HapXIV	GCTTCATGCTACATATTATTGTTTTTCAGCTCTCGAGGGGGCAAACACGCTTGCTNTCGACAAGGGGCCCTT	21	0	0	0

h

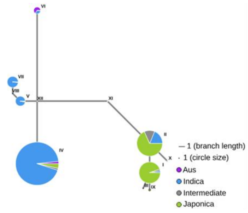

	Haplotype	Indica	Aus	Japonica	Intermediate
HapI	GGTGTATGCAGGGTCCAGGCTCGGATGCTGCCCATTGAAGGCGCGTTTTTGACGTTACAA	22	0	696	19
HapII	GGTGTATGCAGGGTCCAGGCTCGGATGCTGCCCATTGATGGCGCGTTTTTGACGTTACAA	167	0	602	116
HapIII	GGTGTATGCAGGGTCCAGGCTCGGATGCTGCCCATTGAAGGCGCGTTTTTGACGTTACAT	0	0	29	0
HapIV	GGGGCAGATGGGGTCCGGACTCAAGTGTAGNCTGATATAGGCGTGGCCTTGATGCAATAA	1390	27	50	22
HapV	GGGCCAGGNNNNGNNNNNNCTNGGGNNCAACCTGATAAAGGCGTGACCTTGATGCAACAA	326	0	7	1
HapVI	GGTGTGGGTGGGACCTGAAATTGGGCACAGTCTGACAAAGACGTGTTCTTGACTCTTTAA	76	154	1	1
HapVII	GGGCCAGGNNNNGNNNNNNCTNGGGNNCAACCTGATAAAGGCGTGGCCTTGATGCTACAA	374	0	1	3
HapVIII	GGGCCAGGNNNNGNNNNNNCTNGGGNNCAACCTGATAAAGGCGTGACCTTGATGCTACAA	15	0	0	0
HapIX	GGTGTATGCAGGGTCCAGGCTCGGATGCTGCCCATTGAAGGCGCATTTTTGACGTTACAA	0	0	63	0
HapX	GGTGTAGGCGGGGTCCAGGCTCGGATGCTGCCCATTGATGGCGCGTTTTTGACGTTACAA	2	0	19	0
HapXI	GGTGTATGCAGGGTCCAGGCTCGGATGCTGCCCATTGATGGCGTGGCCTTGATGCAATAA	3	32	0	1
HapXII	GGGCCAGGNNNNGNNNNNNNTNGGGNNCAACCTGATAAAGGCGTGGCCTTGATGCAATAA	24	0	1	1

i

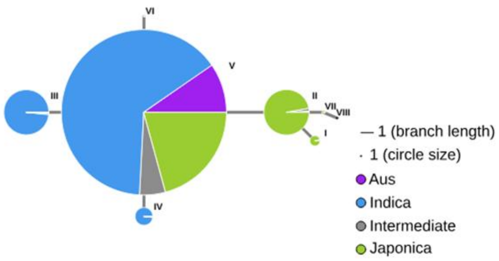

Haplotype		Indica	Aus	Japonica	Intermediate
HapI	CTACGGGCGCCCA	1	0	156	7
HapII	CTACGGGCGCCCG	8	0	709	14
HapIII	AGACGGAAGCCCG	716	0	2	8
HapIV	AGACGGGAGACCG	281	0	1	3
HapV	AGACGGGAGCCCG	1717	258	552	134
HapVI	AGCCGGGAGCCCG	8	11	0	0
HapVII	CTACGGGCCCCCG	1	0	51	2
HapVIII	CTACGGGCCCTCG	0	0	23	0

j

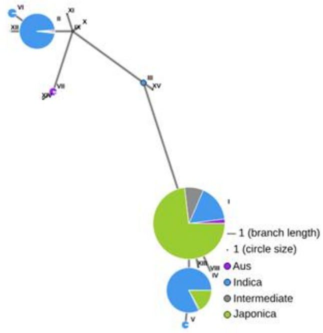

	Haplotype	Indica	Aus	Japonica	Intermediate
HapI	CGATCCCTCTTTGTTCGCGTTGCGGGAGCGTCCGATCCGCTCATCAGT	285	35	1256	146
HapII	CAATCCACCCTTACCCGCATTGCTGGACTATCCGATTTACACTTATGT	824	13	7	8
HapIII	CAATCCATCTTTGTCCGCGTTGCGTGACTACCCAATTCGCTCTTATGT	134	0	0	0
HapIV	CGATCCCTCTTTGTTGGCGTTGCGGGAGCGTCCGATCCGCTCATCAGT	889	0	180	11
HapV	CGATCCCTCTTTGTTGGCGTTGCGGGGGCGTCCGATCCGCTCATCAGT	177	0	0	3
HapVI	CAATCCACCCTTACCCGCATTGCTGCACTATCCGATTTACACTTATGT	215	0	0	1
HapVII	CAATCCACCTCTATCCAAACTGTGGGACTATCCGACTTACACTTATGT	23	160	0	2
HapVIII	CGAAACCTCTTTGTTCGCGTTGCGGGAGCGTCCGATCCGCTCATCAGT	1	0	25	0
HapIX	CAATCCACCCTTATCCGCATTGCGGGACTATCCGATTTACACTTATGT	52	0	0	0
HapX	CATTCCACCCTTATCCGCATTGCGGGACTATCCGATTTACACTTATGT	13	0	1	1
HapXI	CAATCCACCCTTATCCGCATCGCGGGACTATCCGATTTACACTCATGT	13	0	0	0
HapXII	CGATCCACCCTTACCCGCATTGCTGGACTATCCGATTTACACTTATGT	8	26	0	0
HapXIII	CGATCCCTCTTTGTTCGCGTTACGGGAGCGTCCGATCCGCTCATCAGT	0	0	11	0
HapXIV	CAATCCACCTCTATCCAAACTGTGGGACTATCTGACTTACACTTATGT	0	11	0	0
HapXV	CAATCCATCTTTGTCCGCGTTGCGTGACTACTCAATTCGCTCTTATGT	14	0	0	0

k

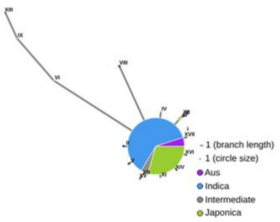

	Haplotype	Indica	Aus	Japonica	Intermediate
HapI	GAGTACCCTGAGGCGGCGCACTCGCGCCAATACATGACCAGACTGTGTCAAGCACTGACTGAAAGAGAGCCCGAGGAG	2265	186	1088	150
HapII	GAGTACCCTGAGGCGGCGCACTCACGCCAATACATGACCAGACTGTGTCAAGCACTGACTGAAAGAGAGCCCGAGGAG	118	0	0	0
HapIII	GAGTACCCTGAGGCGGCACACTCGCGCCAATACATGACCAGACTGTGTCAAGCACTGACTGAAAGAGAGCTCGAGGAG	3	0	223	9
HapIV	GAGTACCCTGAGGCGGCGCATTCGCGCCAATACATGACCAGACTGAGTCAAGCACTGACTGAAAGAGAGCCCGAGGAG	1	0	1	11
HapV	GAGTACCCTGAGGCGGCGCACTCGCGCCAATACATGACCAGACTGTGTCAAGCACTGACTGAAGGAGAGCCCGAGGAG	111	0	0	0
HapVI	ATGAACCTTAAAGCGACGTGCCCGCGTCAACAAATGCCTGGACTATCTTTGGTACCAAGTGAAAAGGTGCCCGGGGTG	2	35	0	0
HapVII	GAGTACCCTGAGGCGGCGCACTCGCGCCAATACATGACCAGGCTGTGTCAAGCACTGACTGAAAGAGAGCCCGAGGAG	1	0	106	1
HapVIII	GAATACCCAGAGGCGGCGCACCCGCGCCTATTCATGCCCGGACTGTCTCAGGCACTGAGAATAAGAATGACCCGAGAG	122	0	0	0
HapIX	ATGTGTCTTAAGGCAGAGTGCCCGCGCCAACAACTACCTGGACTGTCTTTGGCTATAAGTGAAAAAGTGCCAGGGGAG	7	7	0	0
HapX	GAGTACCCTGAGGCGGCACACTCGCGCCAATACATGACCAGACTGTGTCAAGCACTGACTGAGAGAGAGCTCGAGGAG	0	0	12	0
HapXI	GAGTACCCTGAGGCGGCGCACTCGCGCCAATACATGACCAGACTGTGTCAAGCACTGACTGAAAGAGAGCCCGAGGAT	0	20	0	1
HapXII	GAGTACCCTGAGGCGGCACACTCGCGCCAATACATGATCAGACTGTGTCAAGCACTGACTGAAAGAGAGCTCGAGGAG	0	0	13	0
HapXIII	ATGTGCCTTAAGGCGGAGTGCCCGTGCTAACAACCGCCTGGACTGTCTTTGCCTCTAAGTGAAAAAGTACCAGGGGAG	33	0	0	0
HapXIV	GAGTACCCTGAGGCGGCGCACTCGCGCCAATACATGACCAAACTGTGTCAAGCACTGACTGAAAGAGAGCCCGAGGAG	0	0	15	0
HapXV	GAGTACCCTGAGGCGGCGCACTCGCACCAATACATGACCAGGCTGTGTCAAGCACTGACTGAAAGAGAGCCCGAGGAG	0	0	12	0
HapXVI	GAGTACCCTGAGGCGGCGCACTCGCGCCAATACATGACCAGACTGTGTCAAGCACTGACTGAAAGAGAGCCCGAGAAG	11	0	0	0
HapXVII	GAGTACCCTGAGGCGGCGCACTCGCGCCAATACATGACCAGACAGTGTCAAGCACTGACTGAAAGAGAGCCCGAGGAG	13	0	0	0

l

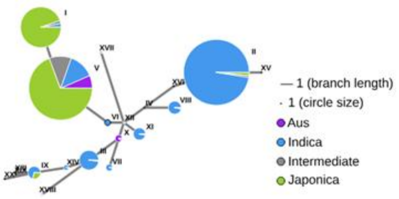

	Haplotype	Indica	Aus	Japonica	Intermediate
HapI	ACGAAGTATTAGCTTTGGGCCTTTCCGGTAAATTA	21	1	648	16
HapII	GCGTAGAATTAACCCAGGGCCGTTCAGGTGAATTA	1084	0	16	10
HapIII	ACGAAGTATCAACCCTGGGCCTTTCAGGAGAATTA	322	0	1	3
HapIV	ACGAAGTATTAACCCTGGGCCGTTCAGGTGAATTA	12	0	0	0
HapV	ACGAAGTATTAGCTCTGGGCCTTTCCGGTAAATTA	140	71	742	121
HapVI	ACGAAGTATTAACCCTGGGCCTTTCCGGTAAATTA	100	0	0	0
HapVII	ACGAAGTATCAACCCTGGGCCGTTCCGGTGGATTA	110	0	0	2
HapVIII	ACGAAGTACTAACCCAGGGCCGTTCAGGTGAATTA	211	0	3	2
HapIX	GCGAAGTATTAACCCTGGGCCTTTCAAGAGAATTA	151	0	59	0
HapX	ACGANGTATCAACCCTGGGCCTTTCCGGTGAATTA	12	104	0	1
HapXI	ACGAAGTATTAACCCTGGGCCTTTCCGGTGGATTA	198	0	0	5
HapXII	ACGAAGTATTAACCCTGGGCCTTTCCGGTGAATTA	43	1	0	2
HapXIII	GCGAAGTATTAACCCTAGGCCTTTCAAGAGAATTA	35	0	0	0
HapXIV	ACGAAGTATTAACCCTGGGCCTTTCAGGAGAATTA	83	0	0	1
HapXV	GCGTAGAATTAACCCAGGGCCGTTCAGGTGAGTTA	11	0	0	0
HapXVI	GCGTAGAATTAACCCTGGGCCGTTCAGGTGAATTA	11	0	0	0
HapXVII	GCGAAATATTAACCCTGGGGTTTCCCGATGAATTA	0	35	0	1
HapXVIII	ATGAAGTATCAACCCTGCCCCTTTCAGGTGAATTA	6	22	6	12
HapXIX	GCGAAGTATTAAACCTGGGCCTTTCAAGAGAATTA	21	0	0	0
HapXX	ACGAAGTATTANCTCTGGGCCTTTCAAGAGAATTA	0	0	12	0

m

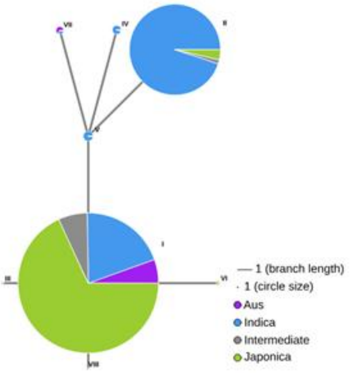

Haplotype		Indica	Aus	Japonica	Intermediate
HapI	AGTATTGGCTCCTGATTTTGACGGGGACTGAGAGCGCGCAGGGGGAACC	386	107	1327	130
HapII	AATATTGGGCCCGGANNNNACAGAGGGGTGNNANTNCGCANNGGNAACC	1202	0	45	18
HapIII	AGTATTGGCTCCTGATTTTGACGGGGACTGAGTGCGCGCAGGGGGAACC	0	0	29	1
HapIV	AGTATTGGATTCTGGNNNNNCAGATAAGTGNNANTNCGCAGNGGGAACC	118	0	3	1
HapV	AGTATTGGGTCCTGANNNNGAAGAGGGGTGNNANNNCGCANNGGNAACC	134	1	0	1
HapVI	AGTATTGGCTCCTGATTTTGACGGGGACTGAGAGCGGTTTGGGGGAACC	0	0	48	4
HapVII	AGTACGCGGTCTTAANNNNGAAGAGGACTGAGAGCGCGCAGTGGGAACC	33	71	0	1
HapVIII	AGTATTGGCTCCTGATTTTGACGGGGACTGAGAGCGCGCAGTGGGAACC	5	29	1	0

n

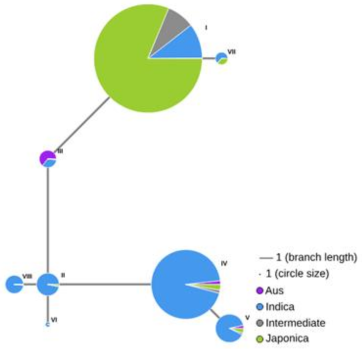

Haplotype		Indica	Aus	Japonica	Intermediate
HapI	CATACACGTGAGATCGCTCCCGGGAA	171	3	1341	137
HapII	CGTACGCTAGNNNNNATTGNCGGAAC	334	0	8	2
HapIII	CATACACGTGAGATCATTCAGGGGGC	89	168	2	1
HapIV	GGAACGCCTGANATCGCTGCCTGAAC	994	19	27	15
HapV	GGAACGCCTGATAGCGCTGCCTGAAC	382	16	22	9
HapVI	CGTACGCTAGATAGCATTGNCGGGAA	72	0	0	1
HapVII	CATACACGTGNGNTNGCTGCCGGGAA	120	0	67	1
HapVIII	CGTATGCTAGATAGCATTGACGGAAC	273	0	0	3

o

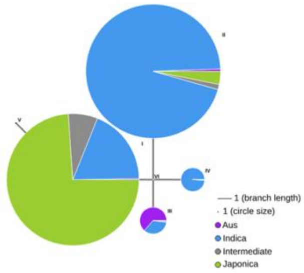

Haplotype		Indica	Aus	Japonica	Intermediate
HapI	CTCCGCAGGCTGG	362	6	1432	139
HapII	CTCCGCTGAATGA	1873	12	61	25
HapIII	CTCCGCTGGCCAG	135	249	2	5
HapIV	CTTCGCTTGCTGG	344	0	0	3
HapV	CTCCCCAGGCTGG	0	0	11	0
HapVI	CTCCGCTGGCTGG	11	0	0	1

Supplementary Fig. 2 Haplotype analysis of 15 potential candidate genes. **a** *LOC_Os02g05260*; **b** LOC_Os12g01140; **c** *LOC_Os12g05440*; **d** *LOC_Os02g05830*; **e** *LOC_Osg12g02040*; **f** *LOC_Osg12g02320*; **g** *LOC_Os02g02780*; **h** *LOC_Os12g02290*; **I** *LOC_Os02g08440*; **j** *LOC_Os02g03410*; **k** *LOC_Os02g03840*; **l** *LOC_Os02g02400*; **m** *LOC_Os12g01370*; **n** *LOC_Os12g02310*; **o** *LOC_Os12g02370*. Circle size corresponds to the number of samples for a given haplotype. Lines between haplotypes indicate mutational steps between alleles. The tables give a summary of the number of haplotypes present in individual genes and their distribution in rice subpopulations. Different colours show the number and or the density of accessions corresponding to subpopulations.

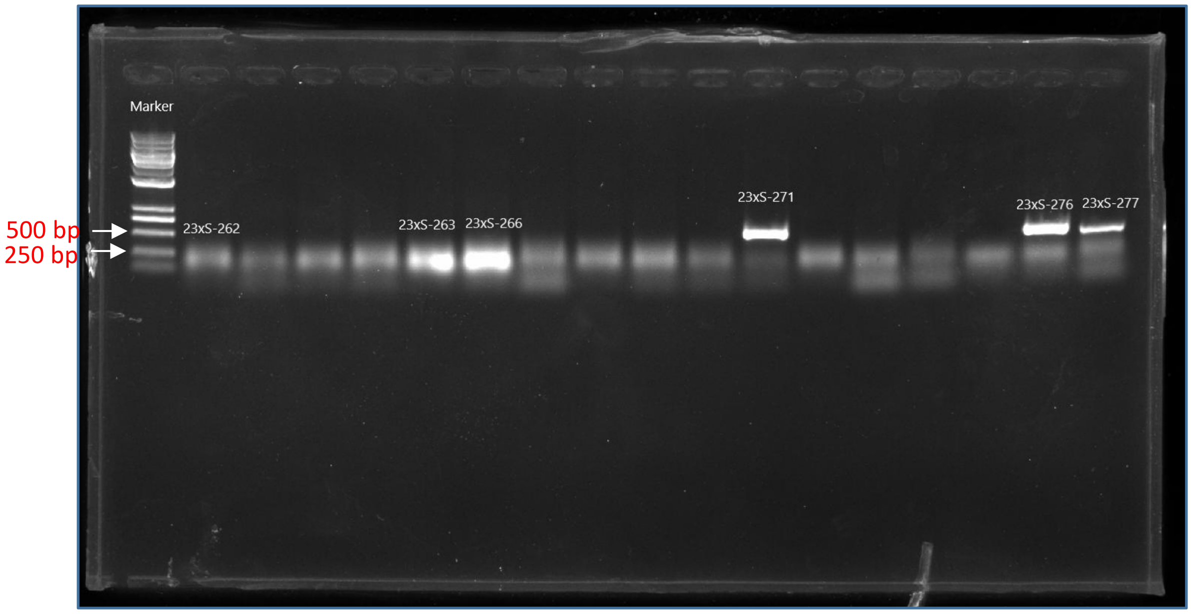

Supplementary Fig. 3 Validation of marker S2329, peak marker of MQTL7.1 on a set of rice genotypes under submerged condition. The allele with 250 bp related to sensitive genotypes and 500 bp allele associated with tolerant genotypes.
